# Translation and validation of a geographic search filter to identify studies about Germany in Embase (Ovid) and MEDLINE(R) ALL (Ovid)

**DOI:** 10.1017/rsm.2025.10016

**Published:** 2025-06-09

**Authors:** Alexander Pachanov, Catharina Muente, Julian Hirt, Dawid Pieper

**Affiliations:** 1 Faculty of Health Sciences Brandenburg, Brandenburg Medical School (Theodor Fontane), Institute for Health Services and Health System Research, Rüdersdorf, Germany; 2 Center for Health Services Research, Brandenburg Medical School (Theodor Fontane), Rüdersdorf, Germany; 3 Pragmatic Evidence Lab, Research Center for Clinical Neuroimmunology and Neuroscience Basel (RC2NB), University Hospital Basel and University of Basel, Basel, Switzerland; 4 Department of Health, Eastern Switzerland University of Applied Sciences, St. Gallen, Switzerland; 5 Institute of Health and Nursing Science, Medical Faculty, Martin Luther University Halle–Wittenberg, Halle (Saale), Germany

**Keywords:** bibliographic databases, Embase, geographic search filters, MEDLINE, Ovid

## Abstract

We developed a geographic search filter for retrieving studies about Germany from PubMed. In this study, we aimed to translate and validate it for use in Embase and MEDLINE(R) ALL via Ovid. Adjustments included aligning PubMed field tags with Ovid’s syntax, adding a keyword heading field for both databases, and incorporating a correspondence address field for Embase. To validate the filters, we used systematic reviews (SRs) that included studies about Germany without imposing geographic restrictions on their search strategies. Subsequently, we conducted (i) case studies (CSs), applying the filters to the search strategies of the 17 eligible SRs; and (ii) aggregation studies, combining the SRs’ search strategies with the ‘OR’ operator and applying the filters. In the CSs, the filters demonstrated a median sensitivity of 100% in both databases, with interquartile ranges (IQRs) of 100%–100% in Embase and 93.75%–100% in MEDLINE(R) ALL. Median precision improved from 0.11% (IQR: 0.05%–0.30%) to 1.65% (IQR: 0.78%–3.06%) and from 0.19% (IQR: 0.11%–0.60%) to 5.13% (IQR: 1.77%–6.85%), while the number needed to read (NNR) decreased from 893.40 (IQR: 354.81–2,219.58) to 60.44 (IQR: 33.94–128.97) and from 513.29 (IQR: 167.35–930.99) to 19.50 (IQR: 14.66–59.35) for Embase and MEDLINE(R) ALL, respectively. In the aggregation studies, the overall sensitivities were 98.19% and 97.14%, with NNRs of 83.29 and 33.34 in Embase and MEDLINE(R) ALL, respectively. The new Embase and MEDLINE(R) ALL filters for Ovid reliably retrieve studies about Germany, enhancing search precision. The approach described in our study can support search filter developers in translating filters for various topics and contexts.

## Highlights

### What is already known

- A validated geographic search filter for MEDLINE (PubMed) to identify studies about Germany was previously developed.
- The filter demonstrated high sensitivity and the potential to reduce workload and resources associated with the screening process.

### What is new

- This work presents validated filters to identify studies about Germany in Embase and MEDLINE(R) ALL via Ovid, translated from the MEDLINE (PubMed) filter.
- The new filters reliably retrieve studies about Germany from both databases.
- Applying both filters together when searching Embase and MEDLINE(R) ALL can help overcome indexing errors and other inconsistencies in cases where the same records are available in both databases.

### Potential impact for RSM readers

- New filters can further support evidence synthesis researchers and other users aiming for comprehensive retrieval of studies about Germany.
- Methodological insights provided in this study can guide search filter developers in translating and validating search filters for other contexts.

## 1 Introduction

Search filters are collections of search terms (single words or groups of words) combined with syntax elements like Boolean operators and field tags, designed to efficiently retrieve records with a common characteristic from bibliographic databases.^1^ ^,^ ^2^ Their validation may involve assessing the performance of search filters in a set of relevant records, either exclusively or in combination with irrelevant records. Validation results enable end-users to compare filter performance and choose the one that best suits their goals, whether prioritizing sensitivity, precision, or a balanced trade-off between the two.^3^

Search filters can be categorized based on their primary purpose. Methodological search filters are designed to identify records of studies with specific research designs (such as randomized controlled trials or systematic reviews [SRs]). In contrast, topic search filters focus on retrieving records related to particular subjects, such as age-related issues, specific diseases, or geographical aspects.^4^

Geographic search filters, a type of topic search filter, are developed to retrieve records with a common geographic focus.^5^ They are particularly useful in evidence syntheses that address research questions sensitive to local contexts. Conducting geographically focused evidence syntheses can be particularly challenging in the absence of such filters.

For instance, a methodological review of search strategies in SRs focusing on health-related studies about Germany highlighted several issues: poorly developed search strategies, incorrect syntax, and the omission of relevant search terms.^6^ The review also emphasized the need for a geographic search filter that reliably retrieves studies about Germany.

In response to these findings and recommendations, we have developed and validated a geographic search filter that identifies studies about Germany in MEDLINE (PubMed).^7^ Our filter was validated on a sample of 178 relevant records and demonstrated a sensitivity of 97.19% and a precision of 2.36%. This filter complements and contributes to the growing collection of validated geographic search filters, which include filters for Spain,^8^ Africa,^9^ the United Kingdom,^2^ ^,^ ^5^ German-speaking countries (specific for high-impact factor nursing journals),^10^ the group of member states of the Organisation for Economic Co-operation and Development,^1^ ^,^ ^11^ and the United States.^12^

While our filter reliably identifies studies about Germany in MEDLINE via PubMed, research indicates that searching multiple databases improves the retrieval of relevant literature, reducing the likelihood of drawing inappropriate conclusions in evidence syntheses.^13^ ^,^ ^14^ In particular, searching Embase was found to be essential for achieving an acceptable level of retrieval of relevant records and should be used in health-related evidence syntheses.^15^

Therefore, we aimed to translate our filter for use in the Embase database (via the Ovid interface). In addition, although PubMed provides free access to MEDLINE, systematic reviewers often prefer using the Ovid interface for MEDLINE searches due to its advanced search capabilities. For example, Ovid offers greater flexibility with proximity operators and truncation, enabling more complex and nuanced search strategies than PubMed.^16^ It also provides a more convenient way to search within abstracts, as Ovid has a dedicated field tag for this purpose, whereas in PubMed, the Title/Abstract field tag must be used, which searches for terms in both the title and abstract rather than the abstract alone. Recognizing these advantages, we also translated our filter for use in MEDLINE(R) ALL via the Ovid interface.

## 2 Methods

In this study, we employed a two-step process to translate and validate our search filters. First, following the approach described in the study on the validation of the Embase UK filter,^2^ we translated the MEDLINE (PubMed) filter to the Ovid interface for Embase and MEDLINE(R) ALL. Second, we validated our filters using the relative recall approach, which allows the validation by utilizing a collection of study records relevant to the filter’s focus, drawn from several comprehensive evidence syntheses.^17^ For validation, we used a sample of 17 SRs that included studies about Germany but did not impose geographical restrictions on their search strategies. Utilizing these SRs, we conducted case studies (CSs) and aggregation studies to calculate the filters’ performance measures. In what follows, we provide more details about each of the study components.

### 2.1 Translation of the MEDLINE (PubMed) filter for use in Embase and MEDLINE(R) ALL via Ovid

The Embase and MEDLINE (R) ALL filters for Ovid were translated from the previously validated MEDLINE (PubMed) filter.^7^ The primary modification involved translating the field tags to align with Ovid’s interface syntax. In addition, two fields were incorporated: the keyword heading (.kw) for both Embase and MEDLINE(R) ALL versions, and the correspondence address, which is available only in the Embase database and was therefore added to the Embase version (Table 1).

**Table 1:** Overview of fields and field tags used for translation of the filter

PubMed field (tag used in the		Ovid MEDLINE(R) ALL field
filter)	Ovid Embase field (tag)	(tag)
Title, abstract, author keywords	Title (.ti)	Title (.ti)
	([Title/Abstract])	Abstract (.ab)	Abstract (.ab)
			Keyword headings (.kw)	Keyword headings (.kw)
MeSH Terms ([MeSH Terms])		Emtree headings (exp *heading*/)	MeSH Terms (exp *MeSH Term*/)
Affiliation ([Affiliation])	Institution (.in)	Institution (.in)
	Correspondence address (.ad)	

*Note*: exp = explode, refers to the process where all the more specific terms listed underneath a given MeSH or Emtree term will be searched; MeSH = Medical Subject Headings.

The (.kw) field was added to Ovid to ensure comprehensive coverage of terms in the PubMed Title/Abstract field, which includes not only the title and abstract but also the keywords provided by authors. We implemented this to ensure consistency with the PubMed filter translation, even though UK filter studies show no benefit in including this field.^2^ ^,^ ^5^ ^,^ ^13^ These studies indicated that the inability to identify records was not due to issues related to the author-provided keywords.^2^ ^,^ ^5^ ^,^ ^13^

An alternative way to cover these keywords would be to use the keyword heading word (.kf), which retrieves every keyword heading that includes a particular word or phrase. However, this field is more likely than (.kw) to retrieve records with keywords referring to products, concepts, or diseases named after German locations but without actual geographic relevance.

For instance, searching with terms from our filter in the (.kf) field can retrieve records with keywords such as *Berlin Questionnaire*, *Nuremberg Argument*, or *Von Recklinghausen disease*. In contrast, searching with the same terms in the (.kw.) field is more precise, retrieving records with keywords that directly correspond to the German cities *Berlin*, *Nuremberg*, and *Recklinghausen*.

Therefore, in our study, the (.kw) field was preferable for maintaining consistency with the PubMed filter while also reducing the retrieval of records containing keywords unrelated to the geographic focus of studies.

### 2.2 Validation of the search filters

#### 2.2.1 Case studies

To validate the translated filters, we utilized the same set of 17 SRs previously employed for validating the MEDLINE (PubMed) filter.^7^ These SRs were used to conduct validation CSs, which involved applying the search filter to the original search strategies of SRs that included studies about Germany and did not geographically restrict their search strategies. Comparing the original search results with those obtained using a search filter enables an evaluation of how much the filter reduces the number of results needing to be screened.

To conduct the CSs, we either reproduced the original search strategies of the SRs when they were available for the Ovid interface or translated them. Since we reused the SRs from our MEDLINE (PubMed) study, where we explicitly searched for SRs with reproducible PubMed search strategies, this resulted in an overrepresentation of PubMed-reported strategies in the current study. Only two SRs reported Ovid search strategies: one for searching Embase and the other for MEDLINE(R) ALL.

When an Ovid search strategy was available for either Embase or MEDLINE(R) ALL, we reproduced the available strategy for the respective database and translated the PubMed strategy for the other database. Five SRs provided search strategies for Embase.com, which we translated for use in Embase Ovid, while translating the PubMed strategy for MEDLINE(R) ALL. When no Ovid search strategies were available for either Embase or MEDLINE(R) ALL, we translated the PubMed strategy for use in both databases via Ovid.

During the translation from MEDLINE (PubMed) to Embase (Ovid), special attention was given to the translation of Medical Subject Headings (MeSH terms) to Emtree terms, as the two databases use different thesauri. To ensure accurate translation, we used the Browse Emtree tool provided by Embase.com.^18^ By entering a MeSH term into the tool, it identifies the corresponding Emtree term. This allows users to explore and verify the correct indexed terms related to their search, ensuring that the terms are accurately aligned with the Emtree indexing system used in Embase.

Since the year of the search was known in all cases, but exact date ranges were not provided by all SRs, we used the entire year as the reference for search dates in all search strategies. We then conducted searches in Embase and MEDLINE(R) ALL via Ovid using the reproduced or translated search strategies of each SR, examining which relevant records identified by the MEDLINE search strategies in PubMed could also be identified in Ovid. Additional details about the search strategies are available in Supplementary Appendix S1.

As a next step, we incorporated the translated version of the filter into the translated or reproduced search strategies using the ‘AND’ Boolean operator. We then compared the search results with and without the filters, to assess their performance in terms of sensitivity, precision, and number needed to read (NNR). The performance measures were defined and calculated as follows:

- **Sensitivity**: the proportion of relevant records identified by a filter version to the total number of relevant records within a set of records, expressed as a percentage^19^:

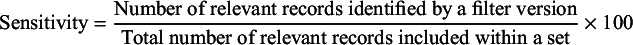

- **Precision**: the proportion of relevant records identified with the search filter to the total number of records identified using the search filter, expressed as a percentage^19^:

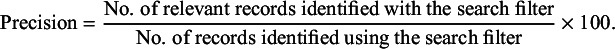

- **NNR**: reflects the number of records needed to be screened to identify a relevant one,^20^ calculated as:

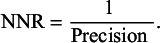

#### 2.2.2 Aggregation studies

In addition to the CSs, we conducted one aggregation study per database using the Ovid interface for both Embase and MEDLINE(R) ALL. This method builds on our earlier work, in which we referred to it as a simulation study during the validation for the MEDLINE (PubMed) search filter to identify studies about Germany.^7^ However, in this work, we use the term *aggregation study* to avoid potential misinterpretation of the term *simulation*, which might suggest computational modeling or artificial data generation, which does not reflect our actual method.

The approach involves developing an aggregated search strategy by combining the search strategies of individual CS SRs with the ‘OR’ Boolean operator to retrieve all relevant records. While a similar approach was used in the post-development study of the NICE UK geographic search filters,^13^ to the best of our knowledge, it has not been applied before for validation purposes.

This method allowed us to compile larger sets of records for validating our filters, providing a broader snapshot of the database and yielding a more representative sample than was obtained from individual CSs. Furthermore, this approach removes duplicate records that would otherwise appear if each search strategy had been run separately. Both these factors help simulate how the filters perform across their respective databases. Consequently, the results from the aggregation study may offer more precise estimates of the filters’ performance measures.

For each database, we then applied the corresponding filter to its respective aggregated search strategy and calculated sensitivity, precision, and NNR.

### 2.3 Analyzing missed records

Based on the results of the CSs and the aggregation studies, we investigated whether all records were identified when applying the filters. In case of missed records, we explored the reasons for their nonretrieval. We reviewed the indexing details of these records, focusing on the presence or absence of Germany-related terms in the records field tags. Furthermore, we examined whether these records were indexed differently across the two databases and whether those overlooked by the Embase filter could be identified using the MEDLINE(R) ALL filter, and vice versa.

## 3 Results

### 3.1 Translation of the MEDLINE (PubMed) filter for use in Embase and MEDLINE(R) ALL via Ovid

The translated search filters are largely identical regarding the fields and the terms used. However, there are two key differences. First, as already mentioned, the Embase filter includes the correspondence address field, which is not available in MEDLINE(R) ALL. Second, the subject headings differ between the two databases. For instance, in Embase, all Germany-related Emtree headings such as ‘German Democratic Republic’, ‘German Federal Republic’, and ‘Prussia’ are organized hierarchically below the Emtree heading ‘Germany’. As a result, searching for the Emtree heading ‘Germany’ retrieves records indexed with these related terms. In contrast, MEDLINE(R) ALL has three MeSH terms for Germany: ‘Germany’ (with the narrower term ‘Berlin’), ‘Germany, West’, and ‘Germany, East’. Unlike ‘Germany’, the latter two do not include narrower terms. Therefore, to ensure comprehensive retrieval of Germany-related records in MEDLINE(R) ALL, a search strategy must explicitly include all three MeSH terms.

Both filters are briefly described in Table 2, while the full filter for Embase can be found in Supplementary Appendix S2 and for MEDLINE(R) ALL in Supplementary Appendix S3.

**Table 2:** Description of the search filters

Embase filter		MEDLINE(R) ALL filter	
Field (tag)	Terms	Field tag	Terms
Title (.ti)	German*	Title (.ti)	German*
Abstract (.ab)	FRG	Abstract (.ab)	FRG
Keyword headings (.kw)	GDR	Keyword headings (.kw)	GDR
Institution (.in)		Institution (.in)	
Correspondence address (.ad)	*Terms related to the names of:*		*Terms related to the names of:*
		Geographical locations		Geographical locations
		Research institutions		Research institutions
		Health insurance funds		Health insurance funds
		Professional organizations		Professional organizations
		Hospitals		Hospitals
Emtree headings (exp *heading*/)	Germany	MeSH Terms (exp *MeSH Term*/)	Germany
						Germany, West
						Germany, East

*Note*: exp = explode, refers to the process where all the more specific terms listed underneath a given MeSH or Emtree term will be searched; FRG = Federal Republic of Germany; GDR = German Democratic Republic; MeSH = Medical Subject Headings.

### 3.2 Validation of the search filters

#### 3.2.1 Case studies

In 17 SRs, the translated or reproduced search strategies identified a total of 166 and 175 relevant records in Embase and MEDLINE(R) ALL, respectively, covering publications from 1981 to 2022 (PMIDs of the records for both databases are presented in Supplementary Appendix S4). Across the SRs, the number of relevant records ranged between 4 and 34 in Embase and between 4 and 36 in MEDLINE(R) ALL.

##### 3.2.1.1 Case studies in Embase (Ovid)

The translated version of the Embase filter demonstrated a median sensitivity of 100% (interquartile range [IQR]: 100%–100%) (Table 3). The maximum sensitivity of 100% was reached in 14 out of 17 CSs, while the sensitivity in the remaining three CSs ranged from 80.00% to 90.91%. Overall, across 17 CSs, three out of 166 records were not identified by the filter. The median precision reached by the filter was 1.65% (IQR: 0.78%–3.06%) and NNR of 60.44 (IQR: 33.94–128.97). Full results of the CSs are available in Supplementary Appendix S5.1.

**Table 3:** Results of the case studies in Embase (Ovid)

	Using search strategies of 17 systematic			Applying translated search filter			
	reviews (without search filter)			to search strategies			
	Hits	Precision	NNR	Hits	Sensitivity	Precision	NNR
		(%)			(%)	(%)	
Median	9,110	0.11	893.40	731	100	1.65	60.44
(IQR)	(2,535–	(0.05–	(354.81–	(231–	(100–	(0.78–	(33.94–
	15,142)	0.30)	2,219.58)	1,148)	100)	3.06)	128.97)

*Note*: IQR = interquartile range; NNR = number needed to read.

##### 3.2.1.2 Case studies in MEDLINE(R) ALL (Ovid)

The MEDLINE(R) ALL filter also demonstrated a median sensitivity of 100% (IQR: 93.75%–100%) (Table 4). In 13 out of 17 CSs, the sensitivity of the filter was 100%, while in the remaining four CSs, it ranged from 83.33% to 87.50%. Out of 175, five records were overlooked by the filter. The filter demonstrated a median precision of 5.13% (IQR: 1.77%–6.85%) and NNR of 19.50 (IQR: 14.66–59.35). Full results of the CSs for MEDLINE(R) ALL are available in Supplementary Appendix S5.2.

**Table 4:** Results of case studies in MEDLINE(R) ALL (Ovid)

	Using search strategies of 17 systematic			Applying translated search filter			
	reviews (without search filter)			to search strategies			
	Hits	Precision	NNR	Hits	Sensitivity	Precision	NNR
Median	4,407	0.19	513.29	261	100	5.13	19.50
(IQR)	(1,323–	(0.11–	(167.35–	(100–	(93.75–	(1.77–	(14.66–
	6,556)	0.60)	930.99)	546)	100)	6.85)	59.35)

*Note*: IQR = interquartile range; NNR = number needed to read.

#### 3.2.2 Aggregation studies

##### 3.2.2.1 Aggregation study in Embase (Ovid)

The aggregated search strategy in Embase retrieved 184,964 records with a precision of 0.09% and an NNR of 1,114.24 (Table 5). Applying the Embase filter reduced the number of hits to 13,577 and improved precision to 1.20%, with NNR of 83.29. The filter identified 163 out of 166 records, achieving a sensitivity of 98.19%.

**Table 5:** Results of the aggregation study in Embase (Ovid)

	Number of hits	Sensitivity (%)	Precision (%)	NNR
Aggregated search strategy	184,964	100	0.09	1,114.24
Translated search filter	13,577	98.19	1.20	83.29

*Note*: NNR = number needed to read.

##### 3.2.2.2 Aggregation study in MEDLINE(R) ALL (Ovid)

The aggregated MEDLINE(R) ALL (Ovid) search strategy demonstrated a precision of 0.21% and an NNR of 486.87, retrieving 85,202 records (Table 6). The application of the translated filter increased precision to 3.00% and decreased NNR to 33.34, while reducing the number of hits to 5,668. The filter identified 170 out of 175 records, achieving a sensitivity of 97.14%.

**Table 6:** Results of the aggregation study in MEDLINE(R) ALL (Ovid)

	Number of hits	Sensitivity (%)	Precision (%)	NNR
Aggregated search strategy	85,202	100	0.21	486.87
Translated search filter	5,668	97.14	3.00	33.34

*Note*: NNR = number needed to read.

### 3.3 Analyzing missed records

Of the three records missed by the Embase filter^21^ ^–^ ^23^ and the five missed by the MEDLINE(R) ALL filter,^22^ ^–^ ^26^ two records^22^ ^,^ ^23^ were not identified by either filter. One record^22^ did not contain terms related to Germany in any field. This record was a report of a multinational study, with the affiliation indexed only for the corresponding author, who was from Australia. In another record,^23^ the only indexed Germany-related term was ‘Germany’ in the Embase ‘country of publication’ field, which was not included in the filter. Notably, the same record was indexed with ‘England’ in the MEDLINE(R) ALL corresponding field and lacked Germany-related terms in any other fields.

Searching for the remaining record missed in Embase^21^ with the MEDLINE(R) ALL filter revealed that it was identifiable through Germany-related terms indexed in the MEDLINE(R) ALL ‘institution’ field. However, in Embase, the record was indexed with terms related to the United States in both the ‘institution’ and ‘correspondence address’ fields.

As for the three remaining records missed by the MEDLINE(R) ALL filter, one record^25^ lacked details in the ‘institution’ field. For the other two records,^24^ ^,^ ^26^ only details on the corresponding author’s institution were provided, which referred to Australia,^24^ and Italy and Spain.^26^ However, all three records were identifiable by the Embase filter through Germany-related terms within the ‘institution’ field indexed for all authors.

## 4 Discussion

In our study, we validated the performance of the filters using two approaches: (1) conducting CSs, which involved calculating the median values of performance measures, and (2) conducting aggregation studies to assess the overall performance using aggregated data from 17 SRs. The CS approach reflected typical filter performance in separate datasets, but did not account for variations in dataset size or the presence of overlapping records. In contrast, the aggregation studies, conducted by combining all search strategies with the ‘OR’ Boolean operator, allowed us to create larger datasets for analysis while removing duplicate records. For example, our aggregated search strategy for Embase retrieved nearly 185,000 unique records. Given that Embase contains ~45.6 million records,^27^ this represents about 0.4% of the total database. Testing the filter on such a sample likely resulted in calculated values that more closely reflect its true performance. By using both approaches, we were able to evaluate the consistency of the filters’ performance in individual datasets and their overall effectiveness in combined datasets. Results from both types of validation approaches applied in our study indicate that the filters reliably retrieve studies about Germany from Embase and MEDLINE(R) ALL using Ovid, achieving a median sensitivity of 100% and an overall sensitivity exceeding 97% in both databases.

Despite reaching high sensitivity, our filters failed to identify three records in Embase and five in MEDLINE(R) ALL. The analysis of these records revealed cases of poor indexing and discrepancies in both databases. For instance, one notable issue was either the complete or partial absence of authors’ affiliation details. This information is particularly important when searching for geographically relevant evidence, as researchers are often more likely to conduct studies in the countries or regions where their institutions are situated.^28^ Without such details, identifying studies about certain geographical locations becomes more challenging. Another issue identified in our study was the inconsistent indexing of author information on country and institutions between Embase and MEDLINE(R) ALL. In one record, the authors were affiliated with German institutions in MEDLINE(R) ALL, but with US institutions in Embase. It is difficult to determine which database contains indexing errors in this case, as researchers from other countries could also conduct research about Germany.

These examples illustrate how inconsistencies in indexing practices across databases can lead to incomplete retrieval of geographically relevant studies. However, our investigation, along with existing research on geographic search filter development,^2^ ^,^ ^13^ suggests that using both filters together during comprehensive literature searches can address such discrepancies and improve the retrieval of relevant records. By doing so, only two records remained unidentified in either database in our study.

Although the filters achieved similar levels of sensitivity in both databases, the MEDLINE(R) ALL filter demonstrated higher precision than the Embase filter. These results align with prior research indicating that searches conducted in Embase tend to yield lower precision compared to those in MEDLINE.^29^ This difference can be attributed to the more extensive indexing in Embase, where records are generally tagged with more subject headings (Emtree terms) than in MEDLINE.^30^ As a result, searches using Emtree terms are more likely to retrieve a larger number of references, including those that may not be relevant, thereby reducing precision. In addition, Embase includes more publication formats than MEDLINE, such as conference abstracts, which contribute to the larger number of results retrieved when searching Embase.

Despite differences in the precision of the filters, their application to the search strategies of the CS SRs consistently improved search precision in both databases, thereby reducing the potential workload associated with abstract screening. Given that abstract screening rates in SRs typically range from 0.13 to 2.88 abstracts per minute,^31^ results of our CSs show the potential for substantial time savings during study selection. The application of the filters resulted in a reduction of the median number of hits by 8,379 in Embase and 4,146 in MEDLINE(R) ALL. At an estimated screening rate of one abstract per minute, this equates to saving ~140 h in Embase and 69 h in MEDLINE(R) ALL. However, it is important to note that this assessment is valid when comparing the performance of our filter to that of search strategies without geographic restrictions—an approach that is uncommon in real-world SRs. As our methodological review shows, 84% of SRs aiming to include studies about Germany applied some form of geographic restriction.^6^ While these restrictions are intended to streamline the identification of relevant studies, we found that they often fail to comprehensively capture all relevant search terms. As a result, such strategies may exclude relevant records while also retrieving fewer results compared to searches without geographic restrictions or those using our filter.

The translation of the filter for use in both Embase and MEDLINE(R) ALL via the Ovid interface offers notable practical benefits for systematic reviewers. The Ovid platform offers advanced search functionalities, including flexible proximity operators and truncation options, which enable the construction of complex search strategies. Furthermore, it has been assessed that translating a search strategy for another database can take up to 79 min.^31^ Conducting searches in Embase and MEDLINE within a single platform, such as Ovid, can help reduce this time by utilizing a shared syntax, common field tags (e.g., title, abstract, author keywords), as well as proximity and truncation operators. This consistency minimizes the need for search strategy adjustments, unlike searches conducted on different platforms where syntax and functionalities often vary.

While a combination of Embase and MEDLINE can capture over 90% of relevant records on health-related topics,^32^ ^,^ ^33^ adding other appropriate databases tailored to the focus of an evidence synthesis is strongly recommended to maximize overall search sensitivity.^15^ We hope that the methods described in this study can assist researchers aiming to conduct comprehensive evidence syntheses with a focus on Germany in translating our filters for use in other databases and search platforms. Automation tools like the Polyglot Search Translator^34^ can assist with this process. While users should be aware of potential errors in search translations when using the Polyglot Search Translator, such errors occur less frequently than with manual methods.^34^ However, it is important to note that Polyglot does not check subject indexing during translation, so users still need to manually verify and adjust MeSH or Emtree terms.

We also hope that our work provides valuable insights into the methodology of translating and validating search filters for search filter developers. A key part of this process is considering differences in database structure, syntax, and indexing practices.

For example, our study shows that translating a search filter from PubMed to the Ovid interface for MEDLINE may be relatively straightforward due to their shared underlying indexing structure. However, challenges can arise when translating Embase filters from the Ovid interface to Embase.com. In Embase.com, the ‘translated title’ field is not automatically included in searches within the ‘title’ field and must be explicitly added as a tag.^35^ This is particularly relevant for geographic search filters that aim to retrieve studies about non-English-speaking countries or regions. Furthermore, as previously mentioned, Embase offers additional fields compared to MEDLINE, which may be useful for translation search filters.

Beyond inconsistencies in search fields, other search functionalities also require attention during translation. While our study did not employ them, proximity and truncation operators may still be useful to developers in this process. Their application varies across databases and search platforms and should be carefully accounted for. In addition, differences in controlled thesauri (e.g., Emtree and MeSH terms) between databases must be addressed to ensure accurate translation.

Once a filter has been translated, the next step is validation to test its reliability and effectiveness. This process can follow the same methodology and use the same record sets from the original filter development and validation. However, if the validation process reveals that amendments to the translated filter are necessary and subsequently made, further validation is required using an independent set of records that were not used during the development or validation.^19^

### 4.1 Strengths and limitations

Given that our filters were translated from the MEDLINE (PubMed) search filter designed to identify studies about Germany,^7^ the strengths and limitations of the original filter also apply to the new filters. The MEDLINE (PubMed) filter was developed using both objective and subjective methodologies for search filter development, described by Jenkins.^19^ A combination of both approaches was also applied during the development of the UK and US filters, whose aim was to maximize sensitivity.^5^ ^,^ ^12^ This approach allowed us to generate a comprehensive set of search terms that consist of the names of German geographical locations, as well as names of professional associations, health insurance funds, and related acronyms. For the Ovid filters, we used the same search terms identified as relevant for the MEDLINE (PubMed) filter, translating them when necessary (e.g., for subject headings in Embase). However, it is important to note that these filters may not cover all relevant search terms.

First, the subjectively developed list of geographical locations included the names of cities with populations of more than 100,000 inhabitants, as well as locations of higher education institutions and university hospitals. This list was supplemented by terms identified through the objective approach. However, some relevant terms, such as the names of smaller cities and towns with populations under 100,000, as well as other terms not identified by either approach, may still not be included in the filters.

Second, we limited the publication dates for records with affiliations to the Federal Republic of Germany (FRG) and German Democratic Republic (GDR) to up to 1990, the year of German reunification. This was done to reduce the number of records retrieved using these terms in the years following reunification, as they could potentially refer to terms unrelated to Germany. We acknowledge that some institutions may have retroactively updated affiliations (e.g., GDR to Germany) after 1990, while articles published after 1990 might still reflect pre-reunification affiliations due to the lag between research completion and publication.

A further potential limitation relates to the validation of the new filters. Our MEDLINE (PubMed) filter was tested on two sets of relevant records before its validation, allowing for amendments to be made during the development to improve its sensitivity. In contrast, the Ovid filters were tested with only one set of records per filter, which was used exclusively for validation. This limited the possibility for amendments, as any changes would have required further validation, necessitating another independent set of records. Generating such a set was particularly challenging in our case, as described in the MEDLINE (PubMed) study.^7^ Identifying SRs eligible for validation CSs required meeting strict criteria: 1) including at least five studies about Germany that were not used in the previous phases, and 2) having a reproducible search strategy that did not have any geographical restrictions. As a result, we were unable to generate an additional set of records for potential further validation. Despite this limitation, the validation results indicated that no amendments were necessary to the search terms used in the filters. The records not identified during validation lacked geographical details related to Germany and were partially retrieved through the complementary use of the filters in both databases.

It is important to mention, however, that one of the missed records^23^ could have been identified in Embase with the term ‘Germany’ if our filter had included the ‘country of publication’ field, which indicates the country where the journal was published. Notably, in MEDLINE(R) ALL, this record is instead indexed with the term ‘England’, further highlighting inconsistencies across databases. However, the decision to not include this field was informed by its effect on precision, as demonstrated during the development of the UK MEDLINE filter.^5^ In that study, excluding the ‘country of publication’ field reduced the number of hits by ~48%. This substantial improvement in precision outweighed the minimal impact on sensitivity, supporting our decision to exclude this field.

The validation results also confirmed that the use of the (.kf) field would not contribute to the performance of the filters in terms of sensitivity. However, we acknowledge that testing filters on larger sets of relevant studies may justify reconsidering the use of the (.kf) field, as it could capture additional relevant records not identified by the terms within (.kw) and other fields applied in our filters.

Certain limitations also arise from the process of translating search strategies between platforms and databases. First, our translated search strategies, which represent the closest possible translation from PubMed, may not fully reflect those that would have been designed specifically for Ovid. Reviewers developing Ovid strategies would likely take advantage of features not available in PubMed, leading to search strategies that yield different results from our translated versions. In addition, differences in controlled vocabulary across databases could further impact search results. For example, the PubMed MeSH terms did not always have identical counterparts in Emtree within Embase. In such cases, we used the Browse Emtree tool to identify the closest equivalent terms. Consequently, these challenges may have influenced the results of our case and aggregation studies, affecting both the total number of records retrieved and the ratio of relevant to nonrelevant records.

## 5 Conclusions

We successfully translated and validated geographic search filters for retrieving studies about Germany from Embase and MEDLINE(R) ALL via the Ovid interface. In the validation studies, the filters demonstrated high sensitivity and the potential to reduce the workload associated with screening and subsequent review steps. Furthermore, using both filters together can further enhance the retrieval of studies about Germany from both databases. We recommend using these filters for researchers aiming to conduct comprehensive evidence syntheses with a focus on Germany. We also hope that our study can support search filter developers and other users in translating and validating search filters for other contexts.

## Supporting information

Pachanov et al. supplementary material Pachanov et al. supplementary material

## Data Availability

The datasets used and/or analyzed during the current study are available in the Supplementary Material.

## Supplementary material

To view supplementary material for this article, please visit http://doi.org/10.1017/rsm.2025.10016.
