# Supplementary material for: Translation and validation of a geographic search filter to identify studies about Germany in Embase (Ovid) and MEDLINE(R) ALL (Ovid)
Source: Res Synth Methods. 2025 Jun 9;16(4):688–700. doi: 10.1017/rsm.2025.10016 (PMC12527497; doi:10.1017/rsm.2025.10016)
Supplement: Pachanov et al. supplementary material [file S1759287925100161sup001.zip › AppendixS4PMIDsOfIncludedStudies.docx]

Appendix S4. PubMed identifiers (PMIDs) of the records of studies about Germany, included in the case and aggregation studies and found with translated or reproduced search strategies.

| **PMIDs of records found with translated or reproduced search strategies in Embase Ovid (n=166)** | **PMIDs of records found with translated or reproduced search strategies in MEDLINE(R) ALL Ovid (n=175)** |
| --- | --- |
| 26324014  23593324  23384407  20472027  19604721  20071233  21108778  17359319  16371880  16133241  32808178  14529334  22384801  22927131  29224062  24372870  25800499  17899899  22092071  25389955  24611678  16244816  18264638  17616343  19924345  22323354  23459981  7798544  9652303  10051707  11240959  11742288  12209104  15144477  16648913  16618364  22030464  25066227  26102348  29654628  11736746  19040466  23306648  27456328  29478012  21262421  24224651  21299292  23631775  16421457  15052512  12817158  15954205  14994321  15323596  25012695  27231151  30028552  30548367  32773397  32708668  33217102  34773420  7942667  7647898  7657042  8590144  8722393  8980933  12055660  20123162  24844107  15298556  11698766  9563707  9692608  12237602  16452833  27855469  26374871  20049465  1915459  4023238  28780766  23761416  7811483  28643593  17310337  28975610  7216575  31437741  34019698  27798749  16552435  16012522  12931015  11694772  11694775  10093610  8374883  16361558  11181656  33200241  22400376  32517203  29649137  25342152  28683852  27178749  15875758  17474848  27428033  20557246  25598040  32813562  26931108  31120316  31277972  11757959  12732887  18289725  26046248  20662616  25611818  16329003  19107970  26338147  19526203  26376038  32445886  33529319  32217063  33931112  17404753  11706328  11791058  10583457  26688509  18092384  24023413  23639825  19781105  19054512  15179129  34410965  22099585  22040695  29143168  31471407  23799905  33590272  23040674  25690923  25458064  17214989  21342499  24747462  27533903  24662853  19565051  30068966  31270555  21914175  25284229  25029437  29396757 | 26324014  25885764  23593324  23384407  20472027  19604721  20071233  21108778  17359319  16371880  16133241  10987709  32808178  14529334  22384801  22927131  29224062  24372870  25800499  17899899  22092071  25389955  24611678  16244816  18264638  17616343  19924345  22323354  23459981  21687611  7798544  9652303  10051707  10565556  11240959  11742288  12209104  15144477  16648913  16618364  22030464  25066227  26102348  29654628  11736746  15479267  23306648  29291478  27456328  29478012  21262421  24224651  23631775  16421457  15052512  12817158  15954205  15323596  25012695  27231151  30028552  30548367  32773397  32708668  33217102  34773420  7942667  7647898  7657042  8590144  8722393  8980933  12055660  10559526  20123162  24844107  15298556  11698766  9563707  9692608  12237602  27855469  26374871  20049465  1915459  4023238  28780766  23761416  7811483  28643593  30944584  17310337  28975610  7216575  31437741  34019698  27798749  16552435  16739325  16012522  12931015  11694772  11694775  10093610  8374883  16361558  11181656  32083990  33200241  22400376  32517203  29649137  25342152  28683852  27178749  15875758  27428033  20557246  25598040  32813562  31120316  31277972  24133543  11757959  12732887  18289725  26046248  20662616  25611818  17456153  16438748  16329003  19107970  26338147  19526203  26376038  32445886  33529319  32217063  33931112  17404753  11706328  11791058  10583457  26688509  18092384  24023413  23639825  19781105  19054512  15179129  34410965  22099585  22040695  29143168  31471407  23799905  33590272  23040674  26815666  25690923  25458064  17214989  21342499  24747462  27533903  24662853  19565051  30068966  31270555  21914175  22249218  25284229  25029437  29396757 |
