# Supplementary material for: Translation and validation of a geographic search filter to identify studies about Germany in Embase (Ovid) and MEDLINE(R) ALL (Ovid)
Source: Res Synth Methods. 2025 Jun 9;16(4):688–700. doi: 10.1017/rsm.2025.10016 (PMC12527497; doi:10.1017/rsm.2025.10016)
Supplement: Pachanov et al. supplementary material [file S1759287925100161sup001.zip › AppendixS5ResultsOfCaseStudies.docx]

Appendix S5.1. Results of the case studies in Embase (Ovid)

|  |  | Using translated or reproduced search strategies of systematic reviews (without search filter) | | | Applying translated search filter to search strategies of systematic reviews | | | | |
| --- | --- | --- | --- | --- | --- | --- | --- | --- | --- |
| First author (year) | No. of relevant records | Hits | Precision (%) | NNR | Hits | Sensitivity (%) | Precision (%) | NNR |  |
| Andrejko (2021) (1) | 10 | 8934 | 0.11 | 893.40 | 596 | 100 | 1.68 | 59.60 |  |
| Cereda (2016) (2) | 9 | 941 | 0.96 | 104.56 | 76 | 100 | 11.84 | 8.44 |  |
| Christensen (2023) (3) | 15 | 9110 | 0.16 | 607.33 | 731 | 100 | 2.05 | 48.73 |  |
| Foley (2022) (4) | 7 | 27634 | 0.03 | 3947.71 | 1743 | 100 | 0.40 | 249.00 |  |
| Gallego (2012) (5) | 6 | 2779 | 0.22 | 463.17 | 243 | 100 | 2.47 | 40.50 |  |
| Gong (2022) (6) | 8 | 2291 | 0.35 | 286.38 | 219 | 100 | 3.65 | 27.38 |  |
| Hu (2017) (7) | 7 | 7497 | 0.09 | 1071.00 | 764 | 85.71 | 0.79 | 127.33 |  |
| Ibrahim (2017) (8) | 8 | 15894 | 0.05 | 1986.75 | 944 | 100 | 0.85 | 118.00 |  |
| Kosmopoulos (2023) (9) | 8 | 21350 | 0.04 | 2668.75 | 962 | 100 | 0.83 | 120.25 |  |
| Lopes (2021) (10) | 8 | 658 | 1.22 | 82.25 | 46 | 100 | 17.39 | 5.75 |  |
| Lu (2023) (11) | 11 | 34526 | 0.03 | 3138.73 | 2479 | 90.91 | 0.40 | 247.90 |  |
| Meng (2022) (12) | 16 | 12411 | 0.13 | 775.69 | 967 | 100 | 1.65 | 60.44 |  |
| Raoofi (2023) (13) | 5 | 5912 | 0.08 | 1182.40 | 420 | 100 | 1.19 | 84.00 |  |
| Singh (2022) (14) | 5 | 9743 | 0.05 | 1948.60 | 653 | 100 | 0.77 | 130.60 |  |
| Ten Cate (2023) (15) | 4 | 416 | 0.96 | 104.00 | 59 | 100 | 6.78 | 14.75 |  |
| Ting (2023) (16) | 34 | 14390 | 0.24 | 423.24 | 1393 | 100 | 2.44 | 40.97 |  |
| Vitturi (2022) (17) | 5 | 12262 | 0.04 | 2452.40 | 1329 | 80.00 | 0.30 | 332.25 |  |
|  | Median  (IQR) | 9110 (2535–15142) | 0.11 (0.05–0.30) | 893.40 (354.81–2219.58) | 731 (231–1148) | 100 (100–100) | 1.65 (0.78–3.06) | 60.44 (33.94–128.97) |  |

NNR = number-needed-to-read
IQR = interquartile range

Appendix S5.2. Results of the case studies in MEDLINE(R) ALL (Ovid)

|  |  | Using translated or reproduced search strategies of systematic reviews (without search filter) | | | Applying translated search filter to search strategies of systematic reviews | | | | |
| --- | --- | --- | --- | --- | --- | --- | --- | --- | --- |
| First author (year) | No. of relevant records | Hits | Precision (%) | NNR | Hits | Sensitivity (%) | Precision (%) | NNR |  |
| Andrejko (2021) (1) | 12 | 4538 | 0.26 | 378.17 | 228 | 100 | 5.26 | 19.00 |  |
| Cereda (2016) (2) | 10 | 510 | 1.96 | 51.00 | 29 | 100 | 34.48 | 2.90 |  |
| Christensen (2023) (3) | 16 | 3622 | 0.44 | 226.38 | 312 | 100 | 5.13 | 19.50 |  |
| Foley (2022) (4) | 7 | 6615 | 0.11 | 945.00 | 494 | 85.71 | 1.21 | 82.33 |  |
| Gallego (2012) (5) | 5 | 1346 | 0.37 | 269.20 | 92 | 100 | 5.43 | 18.40 |  |
| Gong (2022) (6) | 8 | 1299 | 0.62 | 162.38 | 111 | 100 | 7.21 | 13.88 |  |
| Hu (2017) (7) | 7 | 3593 | 0.19 | 513.29 | 234 | 100 | 2.99 | 33.43 |  |
| Ibrahim (2017) (8) | 8 | 11384 | 0.07 | 1423.00 | 577 | 100 | 1.39 | 72.13 |  |
| Kosmopoulos (2023) (9) | 8 | 4406 | 0.18 | 550.88 | 108 | 87.50 | 6.48 | 15.43 |  |
| Lopes (2021) (10) | 8 | 576 | 1.39 | 72.00 | 40 | 100 | 20.00 | 5.00 |  |
| Lu (2023) (11) | 13 | 16564 | 0.08 | 1274.15 | 1061 | 84.62 | 1.04 | 96.45 |  |
| Meng (2022) (12) | 14 | 8962 | 0.16 | 640.14 | 620 | 100 | 2.26 | 44.29 |  |
| Raoofi (2023) (13) | 6 | 4133 | 0.15 | 688.83 | 261 | 100 | 2.30 | 43.50 |  |
| Singh (2022) (14) | 7 | 6497 | 0.11 | 928.14 | 326 | 100 | 2.15 | 46.57 |  |
| Ten Cate (2023) (15) | 4 | 193 | 2.07 | 48.25 | 26 | 100 | 15.38 | 6.50 |  |
| Ting (2023) (16) | 36 | 6203 | 0.58 | 172.31 | 677 | 100 | 5.32 | 18.81 |  |
| Vitturi (2022) (17) | 6 | 5603 | 0.11 | 933.83 | 515 | 83.33 | 0.97 | 103.00 |  |
|  | Median  (IQR) | 4407 (1323–6556) | 0.19 (0.11–0.60) | 513.29 (167.35–930.99) | 261 (100–546) | 100 (93.75–100) | 5.13 (1.77–6.85) | 19.50 (14.66–59.35) |  |

NNR = number-needed-to-read
IQR = interquartile range

## References

1. Andrejko K, Ratnasiri B, Hausdorff WP, Laxminarayan R, Lewnard JA. Antimicrobial resistance in paediatric Streptococcus pneumoniae isolates amid global implementation of pneumococcal conjugate vaccines: a systematic review and meta-regression analysis. Lancet Microbe. 2021;2(9):e450-e60.

2. Cereda E, Pedrolli C, Klersy C, Bonardi C, Quarleri L, Cappello S, et al. Nutritional status in older persons according to healthcare setting: A systematic review and meta-analysis of prevalence data using MNA(®). Clin Nutr. 2016;35(6):1282-90.

3. Christensen MO, Barakji YA, Loft N, Khatib CM, Egeberg A, Thomsen SF, et al. Prevalence of and association between atopic dermatitis and food sensitivity, food allergy and challenge-proven food allergy: A systematic review and meta-analysis. J Eur Acad Dermatol Venereol. 2023;37(5):984-1003.

4. Foley L, Larkin J, Lombard-Vance R, Murphy AW, Hynes L, Galvin E, et al. Prevalence and predictors of medication non-adherence among people living with multimorbidity: a systematic review and meta-analysis. BMJ Open. 2021;11(9):e044987.

5. Gallego JA, Bonetti J, Zhang J, Kane JM, Correll CU. Prevalence and correlates of antipsychotic polypharmacy: a systematic review and meta-regression of global and regional trends from the 1970s to 2009. Schizophr Res. 2012;138(1):18-28.

6. Gong S, Gao Y, Liu J, Li J, Tang X, Ran Q, et al. The prevalence and associated factors of dysphagia in Parkinson's disease: A systematic review and meta-analysis. Front Neurol. 2022;13:1000527.

7. Hu Y, Wan JH, Li XY, Zhu Y, Graham DY, Lu NH. Systematic review with meta-analysis: the global recurrence rate of Helicobacter pylori. Aliment Pharmacol Ther. 2017;46(9):773-9.

8. Ibrahim A, Morais S, Ferro A, Lunet N, Peleteiro B. Sex-differences in the prevalence of Helicobacter pylori infection in pediatric and adult populations: Systematic review and meta-analysis of 244 studies. Dig Liver Dis. 2017;49(7):742-9.

9. Kosmopoulos M, Liatsou Ε, Theochari C, Stavropoulos A, Chatzopoulou D, Mylonas KS, et al. Updates on the Global Prevalence and Etiology of Constrictive Pericarditis: A Systematic Review. Cardiol Rev. 2023.

10. Lopes LB, Machado V, Mascarenhas P, Mendes JJ, Botelho J. The prevalence of molar-incisor hypomineralization: a systematic review and meta-analysis. Sci Rep. 2021;11(1):22405.

11. Lu Y, Pan W, Deng S, Dou Q, Wang X, An Q, et al. Redefining the Incidence and Profile of Fluoropyrimidine-Associated Cardiotoxicity in Cancer Patients: A Systematic Review and Meta-Analysis. Pharmaceuticals (Basel). 2023;16(4).

12. Meng SQ, Cheng JL, Li YY, Yang XQ, Zheng JW, Chang XW, et al. Global prevalence of digital addiction in general population: A systematic review and meta-analysis. Clin Psychol Rev. 2022;92:102128.

13. Raoofi S, Pashazadeh Kan F, Rafiei S, Hosseinipalangi Z, Noorani Mejareh Z, Khani S, et al. Global prevalence of nosocomial infection: A systematic review and meta-analysis. PLoS One. 2023;18(1):e0274248.

14. Singh J, Stoitsova S, Zakrzewska K, Henszel L, Rosińska M, Duffell E. Healthcare-associated hepatitis B and C transmission to patients in the EU/EEA and UK: a systematic review of reported outbreaks between 2006 and 2021. BMC Public Health. 2022;22(1):2260.

15. Ten Cate V, Prochaska JH, Schulz A, Nagler M, Robles AP, Jurk K, et al. Clinical profile and outcome of isolated pulmonary embolism: a systematic review and meta-analysis. EClinicalMedicine. 2023;59:101973.

16. Ting RS, Lewis DP, Yang KX, Nguyen TA, Sarrami P, Daniel L, et al. Incidence of multiple organ failure in adult polytrauma patients: A systematic review and meta-analysis. J Trauma Acute Care Surg. 2023;94(5):725-34.

17. Vitturi BK, Rahmani A, Dini G, Montecucco A, Debarbieri N, Bandiera P, et al. Spatial and temporal distribution of the prevalence of unemployment and early retirement in people with multiple sclerosis: A systematic review with meta-analysis. PLoS One. 2022;17(7):e0272156.
