# Supplementary material for: Translation and validation of a geographic search filter to identify studies about Germany in Embase (Ovid) and MEDLINE(R) ALL (Ovid)
Source: Res Synth Methods. 2025 Jun 9;16(4):688–700. doi: 10.1017/rsm.2025.10016 (PMC12527497; doi:10.1017/rsm.2025.10016)
Supplement: Pachanov et al. supplementary material [file S1759287925100161sup001.zip › AppendixS1SearchStrategies.docx]

Appendix S1. Search strategies of systematic reviews, used to conduct case and aggregation studies

| **Author (year)** | **PubMed search strategy** | **Embase Ovid search strategy** | **MEDLINE(R) ALL Ovid search strategy** |
| --- | --- | --- | --- |
| Andrejko (2021)^1^ | ("Streptococcus pneumoniae"[All Fields] OR (pneumococ[All Fields] OR pneumococaal[All Fields] OR pneumococal[All Fields] OR pneumococc[All Fields] OR pneumococca[All Fields] OR pneumococcaemia[All Fields] OR pneumococcaemic[All Fields] OR pneumococcal[All Fields] OR pneumococcal'[All Fields] OR pneumococcall[All Fields] OR pneumococcalmeningitis[All Fields] OR pneumococcalpneumonia[All Fields] OR pneumococcalvaccine[All Fields] OR pneumococccal[All Fields] OR pneumococcccal[All Fields] OR pneumococcemia[All Fields] OR pneumococcemias[All Fields] OR pneumococcemic[All Fields] OR pneumococcemie[All Fields] OR pneumococcemies[All Fields] OR pneumococchi[All Fields] OR pneumococci[All Fields] OR pneumococci's[All Fields] OR pneumococcia[All Fields] OR pneumococcic[All Fields] OR pneumococcica[All Fields] OR pneumococcicas[All Fields] OR pneumococciche[All Fields] OR pneumococcicidal[All Fields] OR pneumococcicide[All Fields] OR pneumococcico[All Fields] OR pneumococcidal[All Fields] OR pneumococcie[All Fields] OR pneumococcies[All Fields] OR pneumococcique[All Fields] OR pneumococciques[All Fields] OR pneumococcis[All Fields] OR pneumococclea[All Fields] OR pneumococco[All Fields] OR pneumococcoal[All Fields] OR pneumococcol[All Fields] OR pneumococcosis[All Fields] OR pneumococcosuria[All Fields] OR pneumococcous[All Fields] OR pneumococctyper[All Fields] OR pneumococcu[All Fields] OR pneumococcuria[All Fields] OR pneumococcus[All Fields] OR pneumococcus'[All Fields] OR pneumococcus's[All Fields] OR pneumococcus19f[All Fields] OR pneumococcusbetegseg[All Fields] OR pneumococcusclinical[All Fields] OR pneumococcusfertozes[All Fields] OR pneumococcusok[All Fields] OR pneumococcusperitonitis[All Fields] OR pneumococcusprevencio[All Fields] OR pneumococcusuria[All Fields] OR pneumococcusvakcina[All Fields] OR pneumococcusvakcinacio[All Fields] OR pneumococcuswere[All Fields] OR pneumococcusx[All Fields] OR pneumococcy[All Fields] OR pneumococeal[All Fields] OR pneumocochlea[All Fields] OR pneumococi[All Fields] OR pneumococic[All Fields] OR pneumococica[All Fields] OR pneumococicas[All Fields] OR pneumococice[All Fields] OR pneumococicos[All Fields] OR pneumococilor[All Fields] OR pneumococique[All Fields] OR pneumococo[All Fields] OR pneumocococal[All Fields] OR pneumocococcal[All Fields] OR pneumococos[All Fields] OR pneumococul[All Fields] OR pneumococus[All Fields])) AND ((resistan[All Fields] OR resistanc[All Fields] OR resistanca[All Fields] OR resistancce[All Fields] OR resistance[All Fields] OR resistance'[All Fields] OR resistance''[All Fields] OR resistance's[All Fields] OR resistance,[All Fields] OR resistance1[All Fields] OR resistance11[All Fields] OR resistance14[All Fields] OR resistance2[All Fields] OR resistance3[All Fields] OR resistance4[All Fields] OR resistance6[All Fields] OR resistance8[All Fields] OR resistance9[All Fields] OR resistanceamong[All Fields] OR resistanceand[All Fields] OR resistanceare[All Fields] OR resistanceassociated[All Fields] OR resistancebreaking[All Fields] OR resistancecaliber[All Fields] OR resistancecan[All Fields] OR resistanced[All Fields] OR resistancedetermining[All Fields] OR resistancedomaincontaining[All Fields] OR resistancedurability[All Fields] OR resistanceesophageal[All Fields] OR resistanceetween[All Fields] OR resistanceevolution[All Fields] OR resistancega[All Fields] OR resistancein[All Fields] OR resistanceindex[All Fields] OR resistanceir[All Fields] OR resistancekurven[All Fields] OR resistancel[All Fields] OR resistancelocuso[All Fields] OR resistancemap[All Fields] OR resistancemcr[All Fields] OR resistancemediated[All Fields] OR resistancemeter[All Fields] OR resistancenutrition[All Fields] OR resistanceof[All Fields] OR resistanceopen[All Fields] OR resistanceplus[All Fields] OR resistancerate[All Fields] OR resistancerelated[All Fields] OR resistanceright[All Fields] OR resistances[All Fields] OR resistances'[All Fields] OR resistancesim[All Fields] OR resistancesubmitted[All Fields] OR resistancesusceptibility[All Fields] OR resistancethis[All Fields] OR resistanceto[All Fields] OR resistancetype[All Fields] OR resistancevars[All Fields] OR resistancewas[All Fields] OR resistancewere[All Fields] OR resistancewith[All Fields] OR resistancex[All Fields] OR resistancexcompliance[All Fields] OR resistancexpert[All Fields] OR resistancia[All Fields] OR resistancies[All Fields] OR resistanct[All Fields] OR resistancto[All Fields] OR resistancy[All Fields] OR resistand[All Fields] OR resistane[All Fields] OR resistanec[All Fields] OR resistaneza[All Fields] OR resistanfce[All Fields] OR resistange[All Fields] OR resistanhe[All Fields] OR resistanr[All Fields] OR resistans[All Fields] OR resistanse[All Fields] OR resistant[All Fields] OR resistant'[All Fields] OR resistant''[All Fields] OR resistant's[All Fields] OR resistant,[All Fields] OR resistant1[All Fields] OR resistant1's[All Fields] OR resistant11[All Fields] OR resistant12[All Fields] OR resistant2[All Fields] OR resistant2,3[All Fields] OR resistant3[All Fields] OR resistant30[All Fields] OR resistant4[All Fields] OR resistant41[All Fields] OR resistant5[All Fields] OR resistant6[All Fields] OR resistanta[All Fields] OR resistantacid[All Fields] OR resistantacinetobacter[All Fields] OR resistantance[All Fields] OR resistantassociated[All Fields] OR resistantb[All Fields] OR resistantc[All Fields] OR resistantcases[All Fields] OR resistantce[All Fields] OR resistantcell[All Fields] OR resistantcells[All Fields] OR resistante[All Fields] OR resistantenterococci[All Fields] OR resistantenterococcus[All Fields] OR resistantenterococcusdetected[All Fields] OR resistantes[All Fields] OR resistantescherichia[All Fields] OR resistantescherichiacolifrom[All Fields] OR resistantgastric[All Fields] OR resistantgroup[All Fields] OR resistanth[All Fields] OR resistanthypertension[All Fields] OR resistantiae[All Fields] OR resistantin[All Fields] OR resistantl[All Fields] OR resistantlike[All Fields] OR resistantlpr[All Fields] OR resistantly[All Fields] OR resistantm[All Fields] OR resistantmalaria[All Fields] OR resistantmedicago[All Fields] OR resistantmgrbmutants[All Fields] OR resistantmicroorganisms[All Fields] OR resistantmycobacterium[All Fields] OR resistantmycoplasma[All Fields] OR resistantn[All Fields] OR resistantneisseria[All Fields] OR resistantnicotiana[All Fields] OR resistantp[All Fields] OR resistantpathogens[All Fields] OR resistantpathogensidentified[All Fields] OR resistantphenotype[All Fields] OR resistantplants[All Fields] OR resistantplus[All Fields] OR resistantprimary[All Fields] OR resistantproteus[All Fields] OR resistantpseudomonas[All Fields] OR resistantpsuedomonas[All Fields] OR resistantr[All Fields] OR resistants[All Fields] OR resistantstaphylococcus[All Fields] OR resistantstrains[All Fields] OR resistantstreptococcus[All Fields] OR resistanttb[All Fields] OR resistantto[All Fields] OR resistanttodegradationby[All Fields] OR resistanttuberculosis[All Fields] OR resistantu[All Fields] OR resistantwomen[All Fields] OR resistanz[All Fields] OR resistanza[All Fields] OR resistanzce[All Fields] OR resistanzspektrum[All Fields]) OR (susceptib[All Fields] OR susceptibbility[All Fields] OR susceptibble[All Fields] OR susceptibe[All Fields] OR susceptibel[All Fields] OR susceptibiblity[All Fields] OR susceptibie[All Fields] OR susceptibiity[All Fields] OR susceptibil[All Fields] OR susceptibile[All Fields] OR susceptibili[All Fields] OR susceptibilidad[All Fields] OR susceptibilidade[All Fields] OR susceptibilidades[All Fields] OR susceptibilies[All Fields] OR susceptibilify[All Fields] OR susceptibililly[All Fields] OR susceptibililties[All Fields] OR susceptibililty[All Fields] OR susceptibilily[All Fields] OR susceptibilit[All Fields] OR susceptibilita[All Fields] OR susceptibilitat[All Fields] OR susceptibilitate[All Fields] OR susceptibilitatea[All Fields] OR susceptibilitatii[All Fields] OR susceptibilite[All Fields] OR susceptibilites[All Fields] OR susceptibilities[All Fields] OR susceptibilities'[All Fields] OR susceptibilitities[All Fields] OR susceptibilitity[All Fields] OR susceptibilitiy[All Fields] OR susceptibilitv[All Fields] OR susceptibility[All Fields] OR susceptibility'[All Fields] OR susceptibility's[All Fields] OR susceptibility'testing[All Fields] OR susceptibility,[All Fields] OR susceptibility0020might[All Fields] OR susceptibility1[All Fields] OR susceptibility16[All Fields] OR susceptibility2[All Fields] OR susceptibility5[All Fields] OR susceptibilitygene[All Fields] OR susceptibilityof[All Fields] OR susceptibilityrelated[All Fields] OR susceptibilitystatus[All Fields] OR susceptibilityto[All Fields] OR susceptibilitywas[All Fields] OR susceptibilityweighted[All Fields] OR susceptibiliy[All Fields] OR susceptibillities[All Fields] OR susceptibillty[All Fields] OR susceptibilties[All Fields] OR susceptibiltity[All Fields] OR susceptibiltiy[All Fields] OR susceptibilty[All Fields] OR susceptibily[All Fields] OR susceptibitity[All Fields] OR susceptibity[All Fields] OR susceptibiulity[All Fields] OR susceptible[All Fields] OR susceptible'[All Fields] OR susceptible'p[All Fields] OR susceptible1[All Fields] OR susceptible3[All Fields] OR susceptibleinfected[All Fields] OR susceptiblel[All Fields] OR susceptibleleucaena[All Fields] OR susceptiblelycopersicon[All Fields] OR susceptiblemycobacterium[All Fields] OR susceptiblen[All Fields] OR susceptibleness[All Fields] OR susceptibles[All Fields] OR susceptibles'[All Fields] OR susceptiblet[All Fields] OR susceptibleto[All Fields] OR susceptiblexresistant[All Fields] OR susceptibliity[All Fields] OR susceptiblility[All Fields] OR susceptiblilty[All Fields] OR susceptiblities[All Fields] OR susceptiblity[All Fields] OR susceptibly[All Fields]) OR (sensitiv[All Fields] OR sensitiva[All Fields] OR sensitivae[All Fields] OR sensitivas[All Fields] OR sensitivatat[All Fields] OR sensitivation[All Fields] OR sensitivdty[All Fields] OR sensitive[All Fields] OR sensitive'[All Fields] OR sensitive''[All Fields] OR sensitive's[All Fields] OR sensitive1[All Fields] OR sensitive2[All Fields] OR sensitive21[All Fields] OR sensitive23[All Fields] OR sensitive23d[All Fields] OR sensitive3[All Fields] OR sensitive3a[All Fields] OR sensitive4[All Fields] OR sensitive5[All Fields] OR sensitive51[All Fields] OR sensitive51c[All Fields] OR sensitive52[All Fields] OR sensitive81[All Fields] OR sensitive9[All Fields] OR sensitivebiochemical[All Fields] OR sensitivebut[All Fields] OR sensitivecardiac[All Fields] OR sensitived[All Fields] OR sensitivedatura[All Fields] OR sensitiveefflux[All Fields] OR sensitivefsgs[All Fields] OR sensitivehsd[All Fields] OR sensitivelethal[All Fields] OR sensitively[All Fields] OR sensitivem[All Fields] OR sensitivemess[All Fields] OR sensitivemutants[All Fields] OR sensitivemutations[All Fields] OR sensitiven[All Fields] OR sensitivend[All Fields] OR sensitiveness[All Fields] OR sensitivenested[All Fields] OR sensitivepatient[All Fields] OR sensitivepro[All Fields] OR sensitiveprostate[All Fields] OR sensitiver[All Fields] OR sensitives[All Fields] OR sensitives'[All Fields] OR sensitivesensorial[All Fields] OR sensitiveshibire[All Fields] OR sensitiveslow[All Fields] OR sensitivetest[All Fields] OR sensitiveto[All Fields] OR sensitivetoolsforevaluating[All Fields] OR sensitivetu[All Fields] OR sensitivety[All Fields] OR sensitivi[All Fields] OR sensitivi'[All Fields] OR sensitividade[All Fields] OR sensitivie[All Fields] OR sensitiviertem[All Fields] OR sensitivies[All Fields] OR sensitiviity[All Fields] OR sensitivily[All Fields] OR sensitiviness[All Fields] OR sensitiving[All Fields] OR sensitivisation[All Fields] OR sensitivisering[All Fields] OR sensitivit[All Fields] OR sensitivita[All Fields] OR sensitivitas[All Fields] OR sensitivitat[All Fields] OR sensitivitats[All Fields] OR sensitivitatsanalyse[All Fields] OR sensitivitatsanderungen[All Fields] OR sensitivitatsindex[All Fields] OR sensitivitatssteigerung[All Fields] OR sensitivitatsstudie[All Fields] OR sensitivitatsstudien[All Fields] OR sensitivitatstest[All Fields] OR sensitivitatstestung[All Fields] OR sensitivitatsvergleich[All Fields] OR sensitivitatsverlust[All Fields] OR sensitivite[All Fields] OR sensitivites[All Fields] OR sensitivitet[All Fields] OR sensitivitets[All Fields] OR sensitivitetsforvirring[All Fields] OR sensitivitetskurser[All Fields] OR sensitivitetstranas[All Fields] OR sensitivitetstraning[All Fields] OR sensitivitetstraningen[All Fields] OR sensitivitetstrening[All Fields] OR sensitivitiable[All Fields] OR sensitivitied[All Fields] OR sensitivities[All Fields] OR sensitivities'[All Fields] OR sensitivitiesor[All Fields] OR sensitivitiness[All Fields] OR sensitivitites[All Fields] OR sensitivitities[All Fields] OR sensitivitity[All Fields] OR sensitivitive[All Fields] OR sensitivitives[All Fields] OR sensitivitivity[All Fields] OR sensitivitiy[All Fields] OR sensitivitty[All Fields] OR sensitivitv[All Fields] OR sensitivity[All Fields] OR sensitivity'[All Fields] OR sensitivity''[All Fields] OR sensitivity'comt[All Fields] OR sensitivity's[All Fields] OR sensitivity,[All Fields] OR sensitivity0[All Fields] OR sensitivity1[All Fields] OR sensitivity100[All Fields] OR sensitivity26[All Fields] OR sensitivity28[All Fields] OR sensitivity2x2xk[All Fields] OR sensitivity5[All Fields] OR sensitivity60[All Fields] OR sensitivityanalyses[All Fields] OR sensitivityand[All Fields] OR sensitivityc[All Fields] OR sensitivitycalizationand[All Fields] OR sensitivitycardiac[All Fields] OR sensitivitycasecontrol[All Fields] OR sensitivitycontrast[All Fields] OR sensitivityfor[All Fields] OR sensitivityfunction[All Fields] OR sensitivityhits[All Fields] OR sensitivityinconclusive[All Fields] OR sensitivityincreased[All Fields] OR sensitivitykij[All Fields] OR sensitivitylimit[All Fields] OR sensitivitymagnetic[All Fields] OR sensitivitymult[All Fields] OR sensitivityno[All Fields] OR sensitivityof[All Fields] OR sensitivitypowered[All Fields] OR sensitivityr[All Fields] OR sensitivitys[All Fields] OR sensitivitysers[All Fields] OR sensitivityspecificity[All Fields] OR sensitivityspecificitydiagnostic[All Fields] OR sensitivityspecificitypositive[All Fields] OR sensitivitytesting[All Fields] OR sensitivityto[All Fields] OR sensitivitytroponin[All Fields] OR sensitivityvery[All Fields] OR sensitivitywarfarin[All Fields] OR sensitivityxspecificity[All Fields] OR sensitivityxstress[All Fields] OR sensitivive[All Fields] OR sensitiviy[All Fields] OR sensitiviyy[All Fields] OR sensitivization[All Fields] OR sensitivized[All Fields] OR sensitivly[All Fields] OR sensitivnach[All Fields] OR sensitivnaia[All Fields] OR sensitivni[All Fields] OR sensitivniia[All Fields] OR sensitivnogo[All Fields] OR sensitivnom[All Fields] OR sensitivnosti[All Fields] OR sensitivnyi[All Fields] OR sensitivnym[All Fields] OR sensitivnymi[All Fields] OR sensitivo[All Fields] OR sensitivomoteurs[All Fields] OR sensitivomotor[All Fields] OR sensitivomotora[All Fields] OR sensitivomotoras[All Fields] OR sensitivomotrice[All Fields] OR sensitivomotrices[All Fields] OR sensitivomotriz[All Fields] OR sensitivos[All Fields] OR sensitivovegetatif[All Fields] OR sensitivovegetatives[All Fields] OR sensitivrty[All Fields] OR sensitivste[All Fields] OR sensitivt[All Fields] OR sensitivtities[All Fields] OR sensitivtity[All Fields] OR sensitivty[All Fields] OR sensitivum[All Fields] OR sensitivus[All Fields] OR sensitivy[All Fields] OR sensitivyt[All Fields])) AND (carriage[All Fields] OR colonization[All Fields] OR (invasiv[All Fields] OR invasiva[All Fields] OR invasivally[All Fields] OR invasivas[All Fields] OR invasivasui[All Fields] OR invasive[All Fields] OR invasive'[All Fields] OR invasive''[All Fields] OR invasive1[All Fields] OR invasive1,2[All Fields] OR invasive3[All Fields] OR invasive3,4[All Fields] OR invasiveacacia[All Fields] OR invasiveapproach[All Fields] OR invasivebladder[All Fields] OR invasivebrain[All Fields] OR invasivecardiac[All Fields] OR invasivecollection[All Fields] OR invasived[All Fields] OR invasivediagnosis[All Fields] OR invasivedoc[All Fields] OR invasiveescherichia[All Fields] OR invasiveive[All Fields] OR invasiveless[All Fields] OR invasivelesslessbenefit[All Fields] OR invasivelly[All Fields] OR invasively[All Fields] OR invasively'[All Fields] OR invasivem[All Fields] OR invasivemethods[All Fields] OR invasivemodality[All Fields] OR invasivemole[All Fields] OR invasiven[All Fields] OR invasivencss[All Fields] OR invasivene[All Fields] OR invasivenes[All Fields] OR invasiveness[All Fields] OR invasiveness'[All Fields] OR invasivenessdagger[All Fields] OR invasivenesss[All Fields] OR invasivenoninvasive[All Fields] OR invasivenss[All Fields] OR invasiveo[All Fields] OR invasivep[All Fields] OR invasivepossible[All Fields] OR invasiver[All Fields] OR invasiverespiratory[All Fields] OR invasives[All Fields] OR invasives'[All Fields] OR invasivesess[All Fields] OR invasivesness[All Fields] OR invasivespeciesinfo[All Fields] OR invasivesurgery[All Fields] OR invasivetechniques[All Fields] OR invasivetka[All Fields] OR invasivetreatment[All Fields] OR invasivetype[All Fields] OR invasiveusing[All Fields] OR invasivewhile[All Fields] OR invasivi[All Fields] OR invasividad[All Fields] OR invasividade[All Fields] OR invasiviness[All Fields] OR invasivion[All Fields] OR invasivita[All Fields] OR invasivitat[All Fields] OR invasivitatsreduktion[All Fields] OR invasivite[All Fields] OR invasivitet[All Fields] OR invasivities[All Fields] OR invasivity[All Fields] OR invasivkardiologen[All Fields] OR invasivkardiologie[All Fields] OR invasivly[All Fields] OR invasivness[All Fields] OR invasivni[All Fields] OR invasivnykh[All Fields] OR invasivo[All Fields] OR invasivos[All Fields] OR invasivt[All Fields] OR invasivum[All Fields]) OR ("blood"[Subheading] OR "blood"[All Fields] OR "blood"[MeSH Terms]) OR CSF[All Fields] OR (cerebrospin[All Fields] OR cerebrospina[All Fields] OR cerebrospinaie[All Fields] OR cerebrospinaiis[All Fields] OR cerebrospinal[All Fields] OR cerebrospinala[All Fields] OR cerebrospinale[All Fields] OR cerebrospinalen[All Fields] OR cerebrospinaler[All Fields] OR cerebrospinales[All Fields] OR cerebrospinalfluessigkeit[All Fields] OR cerebrospinalfluid[All Fields] OR cerebrospinalflusigkeit[All Fields] OR cerebrospinalflussigkeit[All Fields] OR cerebrospinali[All Fields] OR cerebrospinalis[All Fields] OR cerebrospinalis1[All Fields] OR cerebrospinalisban[All Fields] OR cerebrospinalliquor[All Fields] OR cerebrospinalmeningitt[All Fields] OR cerebrospinalna[All Fields] OR cerebrospinalnaho[All Fields] OR cerebrospinalne[All Fields] OR cerebrospinalneho[All Fields] OR cerebrospinalni[All Fields] OR cerebrospinalniho[All Fields] OR cerebrospinalnim[All Fields] OR cerebrospinalnog[All Fields] OR cerebrospinalnoi[All Fields] OR cerebrospinalnoj[All Fields] OR cerebrospinalnom[All Fields] OR cerebrospinals[All Fields] OR cerebrospinalvaeske[All Fields] OR cerebrospinalvaeskelaekage[All Fields] OR cerebrospinalvaesken[All Fields] OR cerebrospinalvaeskens[All Fields] OR cerebrospinalvatska[All Fields] OR cerebrospinalvatskan[All Fields] OR cerebrospinla[All Fields] OR cerebrospinmal[All Fields] OR cerebrospino[All Fields] OR cerebrospinous[All Fields] OR cerebrospinsl[All Fields] OR cerebrospinslnoj[All Fields]) OR (nasopharyn[All Fields] OR nasopharyneal[All Fields] OR nasopharynectomy[All Fields] OR nasopharynegal[All Fields] OR nasopharynegeal[All Fields] OR nasopharyng[All Fields] OR nasopharyngaeal[All Fields] OR nasopharyngael[All Fields] OR nasopharyngaitis[All Fields] OR nasopharyngal[All Fields] OR nasopharynge[All Fields] OR nasopharyngea[All Fields] OR nasopharyngead[All Fields] OR nasopharyngeal[All Fields] OR nasopharyngeal'[All Fields] OR nasopharyngealbordetella[All Fields] OR nasopharyngealcarcinoma[All Fields] OR nasopharyngeale[All Fields] OR nasopharyngealelektroden[All Fields] OR nasopharyngealem[All Fields] OR nasopharyngealen[All Fields] OR nasopharyngealer[All Fields] OR nasopharyngeales[All Fields] OR nasopharyngealis[All Fields] OR nasopharyngeally[All Fields] OR nasopharyngealpassages[All Fields] OR nasopharyngealpodning[All Fields] OR nasopharyngealt[All Fields] OR nasopharyngeat[All Fields] OR nasopharyngectomies[All Fields] OR nasopharyngectomy[All Fields] OR nasopharyngee[All Fields] OR nasopharyngel[All Fields] OR nasopharyngeoscopy[All Fields] OR nasopharyngerl[All Fields] OR nasopharynges[All Fields] OR nasopharyngeum[All Fields] OR nasopharyngeus[All Fields] OR nasopharyngheal[All Fields] OR nasopharyngial[All Fields] OR nasopharyngien[All Fields] OR nasopharyngienne[All Fields] OR nasopharyngiens[All Fields] OR nasopharyngioma[All Fields] OR nasopharyngis[All Fields] OR nasopharyngital[All Fields] OR nasopharyngitidis[All Fields] OR nasopharyngitis[All Fields] OR nasopharyngitis'[All Fields] OR nasopharyngitises[All Fields] OR nasopharyngo[All Fields] OR nasopharyngoal[All Fields] OR nasopharyngobronchial[All Fields] OR nasopharyngoendoscopy[All Fields] OR nasopharyngofiberoscope[All Fields] OR nasopharyngofiberscopy[All Fields] OR nasopharyngogram[All Fields] OR nasopharyngograms[All Fields] OR nasopharyngography[All Fields] OR nasopharyngolarygnoscope[All Fields] OR nasopharyngolaryngeal[All Fields] OR nasopharyngolaryngee[All Fields] OR nasopharyngolaryngoscope[All Fields] OR nasopharyngolaryngoscopes[All Fields] OR nasopharyngolaryngoscopic[All Fields] OR nasopharyngolaryngoscopie[All Fields] OR nasopharyngolaryngoscopies[All Fields] OR nasopharyngolaryngoscopy[All Fields] OR nasopharyngolaryngovideoscope[All Fields] OR nasopharyngolarynx[All Fields] OR nasopharyngometry[All Fields] OR nasopharyngoscope[All Fields] OR nasopharyngoscopes[All Fields] OR nasopharyngoscopic[All Fields] OR nasopharyngoscopically[All Fields] OR nasopharyngoscopies[All Fields] OR nasopharyngoscopy[All Fields] OR nasopharyngoskop[All Fields] OR nasopharyngosopic[All Fields] OR nasopharyngotis[All Fields] OR nasopharyngral[All Fields] OR nasopharyngs[All Fields] OR nasopharyngscopy[All Fields] OR nasopharyngtis[All Fields] OR nasopharyngus[All Fields] OR nasopharyngx[All Fields] OR nasopharynlaryngoscope[All Fields] OR nasopharynogoscopy[All Fields] OR nasopharyns[All Fields] OR nasopharynx[All Fields] OR nasopharynx'[All Fields] OR nasopharynx's[All Fields] OR nasopharynx2[All Fields] OR nasopharynxandnasopharyngeal[All Fields] OR nasopharynxaspirat[All Fields] OR nasopharynxbefall[All Fields] OR nasopharynxduring[All Fields] OR nasopharynxelektrode[All Fields] OR nasopharynxes[All Fields] OR nasopharynxkarcinomer[All Fields] OR nasopharynxkarzinom[All Fields] OR nasopharynxkarzinome[All Fields] OR nasopharynxkarzinomen[All Fields] OR nasopharynxkarzinompatienten[All Fields] OR nasopharynxkarzinoms[All Fields] OR nasopharynxmalignom[All Fields] OR nasopharynxmalignome[All Fields] OR nasopharynxprozessen[All Fields] OR nasopharynxtuberkulos[All Fields] OR nasopharynxtuberkulose[All Fields] OR nasopharynxtumor[All Fields] OR nasopharynxtumoren[All Fields] OR nasopharynxtumors[All Fields] OR nasopharyny[All Fields] OR nasopharynyeal[All Fields] OR nasopharynz[All Fields])) AND ("2000/01/01"[PDAT] : "2020/11/24"[PDAT]) | ("Streptococcus pneumoniae".af. OR (pneumococ.af. OR pneumococaal.af. OR pneumococal.af. OR pneumococc.af. OR pneumococca.af. OR pneumococcaemia.af. OR pneumococcaemic.af. OR pneumococcal.af. OR pneumococcal'.af. OR pneumococcall.af. OR pneumococcalmeningitis.af. OR pneumococcalpneumonia.af. OR pneumococcalvaccine.af. OR pneumococccal.af. OR pneumococcccal.af. OR pneumococcemia.af. OR pneumococcemias.af. OR pneumococcemic.af. OR pneumococcemie.af. OR pneumococcemies.af. OR pneumococchi.af. OR pneumococci.af. OR pneumococci's.af. OR pneumococcia.af. OR pneumococcic.af. OR pneumococcica.af. OR pneumococcicas.af. OR pneumococciche.af. OR pneumococcicidal.af. OR pneumococcicide.af. OR pneumococcico.af. OR pneumococcidal.af. OR pneumococcie.af. OR pneumococcies.af. OR pneumococcique.af. OR pneumococciques.af. OR pneumococcis.af. OR pneumococclea.af. OR pneumococco.af. OR pneumococcoal.af. OR pneumococcol.af. OR pneumococcosis.af. OR pneumococcosuria.af. OR pneumococcous.af. OR pneumococctyper.af. OR pneumococcu.af. OR pneumococcuria.af. OR pneumococcus.af. OR pneumococcus'.af. OR pneumococcus's.af. OR pneumococcus19f.af. OR pneumococcusbetegseg.af. OR pneumococcusclinical.af. OR pneumococcusfertozes.af. OR pneumococcusok.af. OR pneumococcusperitonitis.af. OR pneumococcusprevencio.af. OR pneumococcusuria.af. OR pneumococcusvakcina.af. OR pneumococcusvakcinacio.af. OR pneumococcuswere.af. OR pneumococcusx.af. OR pneumococcy.af. OR pneumococeal.af. OR pneumocochlea.af. OR pneumococi.af. OR pneumococic.af. OR pneumococica.af. OR pneumococicas.af. OR pneumococice.af. OR pneumococicos.af. OR pneumococilor.af. OR pneumococique.af. OR pneumococo.af. OR pneumocococal.af. OR pneumocococcal.af. OR pneumococos.af. OR pneumococul.af. OR pneumococus.af.)) AND ((resistan.af. OR resistanc.af. OR resistanca.af. OR resistancce.af. OR resistance.af. OR resistance'.af. OR resistance''.af. OR resistance's.af. OR resistance,.af. OR resistance1.af. OR resistance11.af. OR resistance14.af. OR resistance2.af. OR resistance3.af. OR resistance4.af. OR resistance6.af. OR resistance8.af. OR resistance9.af. OR resistanceamong.af. OR resistanceand.af. OR resistanceare.af. OR resistanceassociated.af. OR resistancebreaking.af. OR resistancecaliber.af. OR resistancecan.af. OR resistanced.af. OR resistancedetermining.af. OR resistancedomaincontaining.af. OR resistancedurability.af. OR resistanceesophageal.af. OR resistanceetween.af. OR resistanceevolution.af. OR resistancega.af. OR resistancein.af. OR resistanceindex.af. OR resistanceir.af. OR resistancekurven.af. OR resistancel.af. OR resistancelocuso.af. OR resistancemap.af. OR resistancemcr.af. OR resistancemediated.af. OR resistancemeter.af. OR resistancenutrition.af. OR resistanceof.af. OR resistanceopen.af. OR resistanceplus.af. OR resistancerate.af. OR resistancerelated.af. OR resistanceright.af. OR resistances.af. OR resistances'.af. OR resistancesim.af. OR resistancesubmitted.af. OR resistancesusceptibility.af. OR resistancethis.af. OR resistanceto.af. OR resistancetype.af. OR resistancevars.af. OR resistancewas.af. OR resistancewere.af. OR resistancewith.af. OR resistancex.af. OR resistancexcompliance.af. OR resistancexpert.af. OR resistancia.af. OR resistancies.af. OR resistanct.af. OR resistancto.af. OR resistancy.af. OR resistand.af. OR resistane.af. OR resistanec.af. OR resistaneza.af. OR resistanfce.af. OR resistange.af. OR resistanhe.af. OR resistanr.af. OR resistans.af. OR resistanse.af. OR resistant.af. OR resistant'.af. OR resistant''.af. OR resistant's.af. OR resistant,.af. OR resistant1.af. OR resistant1's.af. OR resistant11.af. OR resistant12.af. OR resistant2.af. OR resistant2,3.af. OR resistant3.af. OR resistant30.af. OR resistant4.af. OR resistant41.af. OR resistant5.af. OR resistant6.af. OR resistanta.af. OR resistantacid.af. OR resistantacinetobacter.af. OR resistantance.af. OR resistantassociated.af. OR resistantb.af. OR resistantc.af. OR resistantcases.af. OR resistantce.af. OR resistantcell.af. OR resistantcells.af. OR resistante.af. OR resistantenterococci.af. OR resistantenterococcus.af. OR resistantenterococcusdetected.af. OR resistantes.af. OR resistantescherichia.af. OR resistantescherichiacolifrom.af. OR resistantgastric.af. OR resistantgroup.af. OR resistanth.af. OR resistanthypertension.af. OR resistantiae.af. OR resistantin.af. OR resistantl.af. OR resistantlike.af. OR resistantlpr.af. OR resistantly.af. OR resistantm.af. OR resistantmalaria.af. OR resistantmedicago.af. OR resistantmgrbmutants.af. OR resistantmicroorganisms.af. OR resistantmycobacterium.af. OR resistantmycoplasma.af. OR resistantn.af. OR resistantneisseria.af. OR resistantnicotiana.af. OR resistantp.af. OR resistantpathogens.af. OR resistantpathogensidentified.af. OR resistantphenotype.af. OR resistantplants.af. OR resistantplus.af. OR resistantprimary.af. OR resistantproteus.af. OR resistantpseudomonas.af. OR resistantpsuedomonas.af. OR resistantr.af. OR resistants.af. OR resistantstaphylococcus.af. OR resistantstrains.af. OR resistantstreptococcus.af. OR resistanttb.af. OR resistantto.af. OR resistanttodegradationby.af. OR resistanttuberculosis.af. OR resistantu.af. OR resistantwomen.af. OR resistanz.af. OR resistanza.af. OR resistanzce.af. OR resistanzspektrum.af.) OR (susceptib.af. OR susceptibbility.af. OR susceptibble.af. OR susceptibe.af. OR susceptibel.af. OR susceptibiblity.af. OR susceptibie.af. OR susceptibiity.af. OR susceptibil.af. OR susceptibile.af. OR susceptibili.af. OR susceptibilidad.af. OR susceptibilidade.af. OR susceptibilidades.af. OR susceptibilies.af. OR susceptibilify.af. OR susceptibililly.af. OR susceptibililties.af. OR susceptibililty.af. OR susceptibilily.af. OR susceptibilit.af. OR susceptibilita.af. OR susceptibilitat.af. OR susceptibilitate.af. OR susceptibilitatea.af. OR susceptibilitatii.af. OR susceptibilite.af. OR susceptibilites.af. OR susceptibilities.af. OR susceptibilities'.af. OR susceptibilitities.af. OR susceptibilitity.af. OR susceptibilitiy.af. OR susceptibilitv.af. OR susceptibility.af. OR susceptibility'.af. OR susceptibility's.af. OR susceptibility'testing.af. OR susceptibility,.af. OR susceptibility0020might.af. OR susceptibility1.af. OR susceptibility16.af. OR susceptibility2.af. OR susceptibility5.af. OR susceptibilitygene.af. OR susceptibilityof.af. OR susceptibilityrelated.af. OR susceptibilitystatus.af. OR susceptibilityto.af. OR susceptibilitywas.af. OR susceptibilityweighted.af. OR susceptibiliy.af. OR susceptibillities.af. OR susceptibillty.af. OR susceptibilties.af. OR susceptibiltity.af. OR susceptibiltiy.af. OR susceptibilty.af. OR susceptibily.af. OR susceptibitity.af. OR susceptibity.af. OR susceptibiulity.af. OR susceptible.af. OR susceptible'.af. OR susceptible'p.af. OR susceptible1.af. OR susceptible3.af. OR susceptibleinfected.af. OR susceptiblel.af. OR susceptibleleucaena.af. OR susceptiblelycopersicon.af. OR susceptiblemycobacterium.af. OR susceptiblen.af. OR susceptibleness.af. OR susceptibles.af. OR susceptibles'.af. OR susceptiblet.af. OR susceptibleto.af. OR susceptiblexresistant.af. OR susceptibliity.af. OR susceptiblility.af. OR susceptiblilty.af. OR susceptiblities.af. OR susceptiblity.af. OR susceptibly.af.) OR (sensitiv.af. OR sensitiva.af. OR sensitivae.af. OR sensitivas.af. OR sensitivatat.af. OR sensitivation.af. OR sensitivdty.af. OR sensitive.af. OR sensitive'.af. OR sensitive''.af. OR sensitive's.af. OR sensitive1.af. OR sensitive2.af. OR sensitive21.af. OR sensitive23.af. OR sensitive23d.af. OR sensitive3.af. OR sensitive3a.af. OR sensitive4.af. OR sensitive5.af. OR sensitive51.af. OR sensitive51c.af. OR sensitive52.af. OR sensitive81.af. OR sensitive9.af. OR sensitivebiochemical.af. OR sensitivebut.af. OR sensitivecardiac.af. OR sensitived.af. OR sensitivedatura.af. OR sensitiveefflux.af. OR sensitivefsgs.af. OR sensitivehsd.af. OR sensitivelethal.af. OR sensitively.af. OR sensitivem.af. OR sensitivemess.af. OR sensitivemutants.af. OR sensitivemutations.af. OR sensitiven.af. OR sensitivend.af. OR sensitiveness.af. OR sensitivenested.af. OR sensitivepatient.af. OR sensitivepro.af. OR sensitiveprostate.af. OR sensitiver.af. OR sensitives.af. OR sensitives'.af. OR sensitivesensorial.af. OR sensitiveshibire.af. OR sensitiveslow.af. OR sensitivetest.af. OR sensitiveto.af. OR sensitivetoolsforevaluating.af. OR sensitivetu.af. OR sensitivety.af. OR sensitivi.af. OR sensitivi'.af. OR sensitividade.af. OR sensitivie.af. OR sensitiviertem.af. OR sensitivies.af. OR sensitiviity.af. OR sensitivily.af. OR sensitiviness.af. OR sensitiving.af. OR sensitivisation.af. OR sensitivisering.af. OR sensitivit.af. OR sensitivita.af. OR sensitivitas.af. OR sensitivitat.af. OR sensitivitats.af. OR sensitivitatsanalyse.af. OR sensitivitatsanderungen.af. OR sensitivitatsindex.af. OR sensitivitatssteigerung.af. OR sensitivitatsstudie.af. OR sensitivitatsstudien.af. OR sensitivitatstest.af. OR sensitivitatstestung.af. OR sensitivitatsvergleich.af. OR sensitivitatsverlust.af. OR sensitivite.af. OR sensitivites.af. OR sensitivitet.af. OR sensitivitets.af. OR sensitivitetsforvirring.af. OR sensitivitetskurser.af. OR sensitivitetstranas.af. OR sensitivitetstraning.af. OR sensitivitetstraningen.af. OR sensitivitetstrening.af. OR sensitivitiable.af. OR sensitivitied.af. OR sensitivities.af. OR sensitivities'.af. OR sensitivitiesor.af. OR sensitivitiness.af. OR sensitivitites.af. OR sensitivitities.af. OR sensitivitity.af. OR sensitivitive.af. OR sensitivitives.af. OR sensitivitivity.af. OR sensitivitiy.af. OR sensitivitty.af. OR sensitivitv.af. OR sensitivity.af. OR sensitivity'.af. OR sensitivity''.af. OR sensitivity'comt.af. OR sensitivity's.af. OR sensitivity,.af. OR sensitivity0.af. OR sensitivity1.af. OR sensitivity100.af. OR sensitivity26.af. OR sensitivity28.af. OR sensitivity2x2xk.af. OR sensitivity5.af. OR sensitivity60.af. OR sensitivityanalyses.af. OR sensitivityand.af. OR sensitivityc.af. OR sensitivitycalizationand.af. OR sensitivitycardiac.af. OR sensitivitycasecontrol.af. OR sensitivitycontrast.af. OR sensitivityfor.af. OR sensitivityfunction.af. OR sensitivityhits.af. OR sensitivityinconclusive.af. OR sensitivityincreased.af. OR sensitivitykij.af. OR sensitivitylimit.af. OR sensitivitymagnetic.af. OR sensitivitymult.af. OR sensitivityno.af. OR sensitivityof.af. OR sensitivitypowered.af. OR sensitivityr.af. OR sensitivitys.af. OR sensitivitysers.af. OR sensitivityspecificity.af. OR sensitivityspecificitydiagnostic.af. OR sensitivityspecificitypositive.af. OR sensitivitytesting.af. OR sensitivityto.af. OR sensitivitytroponin.af. OR sensitivityvery.af. OR sensitivitywarfarin.af. OR sensitivityxspecificity.af. OR sensitivityxstress.af. OR sensitivive.af. OR sensitiviy.af. OR sensitiviyy.af. OR sensitivization.af. OR sensitivized.af. OR sensitivly.af. OR sensitivnach.af. OR sensitivnaia.af. OR sensitivni.af. OR sensitivniia.af. OR sensitivnogo.af. OR sensitivnom.af. OR sensitivnosti.af. OR sensitivnyi.af. OR sensitivnym.af. OR sensitivnymi.af. OR sensitivo.af. OR sensitivomoteurs.af. OR sensitivomotor.af. OR sensitivomotora.af. OR sensitivomotoras.af. OR sensitivomotrice.af. OR sensitivomotrices.af. OR sensitivomotriz.af. OR sensitivos.af. OR sensitivovegetatif.af. OR sensitivovegetatives.af. OR sensitivrty.af. OR sensitivste.af. OR sensitivt.af. OR sensitivtities.af. OR sensitivtity.af. OR sensitivty.af. OR sensitivum.af. OR sensitivus.af. OR sensitivy.af. OR sensitivyt.af.)) AND (carriage.af. OR colonization.af. OR (invasiv.af. OR invasiva.af. OR invasivally.af. OR invasivas.af. OR invasivasui.af. OR invasive.af. OR invasive'.af. OR invasive''.af. OR invasive1.af. OR invasive1,2.af. OR invasive3.af. OR invasive3,4.af. OR invasiveacacia.af. OR invasiveapproach.af. OR invasivebladder.af. OR invasivebrain.af. OR invasivecardiac.af. OR invasivecollection.af. OR invasived.af. OR invasivediagnosis.af. OR invasivedoc.af. OR invasiveescherichia.af. OR invasiveive.af. OR invasiveless.af. OR invasivelesslessbenefit.af. OR invasivelly.af. OR invasively.af. OR invasively'.af. OR invasivem.af. OR invasivemethods.af. OR invasivemodality.af. OR invasivemole.af. OR invasiven.af. OR invasivencss.af. OR invasivene.af. OR invasivenes.af. OR invasiveness.af. OR invasiveness'.af. OR invasivenessdagger.af. OR invasivenesss.af. OR invasivenoninvasive.af. OR invasivenss.af. OR invasiveo.af. OR invasivep.af. OR invasivepossible.af. OR invasiver.af. OR invasiverespiratory.af. OR invasives.af. OR invasives'.af. OR invasivesess.af. OR invasivesness.af. OR invasivespeciesinfo.af. OR invasivesurgery.af. OR invasivetechniques.af. OR invasivetka.af. OR invasivetreatment.af. OR invasivetype.af. OR invasiveusing.af. OR invasivewhile.af. OR invasivi.af. OR invasividad.af. OR invasividade.af. OR invasiviness.af. OR invasivion.af. OR invasivita.af. OR invasivitat.af. OR invasivitatsreduktion.af. OR invasivite.af. OR invasivitet.af. OR invasivities.af. OR invasivity.af. OR invasivkardiologen.af. OR invasivkardiologie.af. OR invasivly.af. OR invasivness.af. OR invasivni.af. OR invasivnykh.af. OR invasivo.af. OR invasivos.af. OR invasivt.af. OR invasivum.af.) OR ("Blood".fs. OR blood.af. OR exp blood/) OR CSF.af. OR (cerebrospin.af. OR cerebrospina.af. OR cerebrospinaie.af. OR cerebrospinaiis.af. OR cerebrospinal.af. OR cerebrospinala.af. OR cerebrospinale.af. OR cerebrospinalen.af. OR cerebrospinaler.af. OR cerebrospinales.af. OR cerebrospinalfluessigkeit.af. OR cerebrospinalfluid.af. OR cerebrospinalflusigkeit.af. OR cerebrospinalflussigkeit.af. OR cerebrospinali.af. OR cerebrospinalis.af. OR cerebrospinalis1.af. OR cerebrospinalisban.af. OR cerebrospinalliquor.af. OR cerebrospinalmeningitt.af. OR cerebrospinalna.af. OR cerebrospinalnaho.af. OR cerebrospinalne.af. OR cerebrospinalneho.af. OR cerebrospinalni.af. OR cerebrospinalniho.af. OR cerebrospinalnim.af. OR cerebrospinalnog.af. OR cerebrospinalnoi.af. OR cerebrospinalnoj.af. OR cerebrospinalnom.af. OR cerebrospinals.af. OR cerebrospinalvaeske.af. OR cerebrospinalvaeskelaekage.af. OR cerebrospinalvaesken.af. OR cerebrospinalvaeskens.af. OR cerebrospinalvatska.af. OR cerebrospinalvatskan.af. OR cerebrospinla.af. OR cerebrospinmal.af. OR cerebrospino.af. OR cerebrospinous.af. OR cerebrospinsl.af. OR cerebrospinslnoj.af.) OR (nasopharyn.af. OR nasopharyneal.af. OR nasopharynectomy.af. OR nasopharynegal.af. OR nasopharynegeal.af. OR nasopharyng.af. OR nasopharyngaeal.af. OR nasopharyngael.af. OR nasopharyngaitis.af. OR nasopharyngal.af. OR nasopharynge.af. OR nasopharyngea.af. OR nasopharyngead.af. OR nasopharyngeal.af. OR nasopharyngeal'.af. OR nasopharyngealbordetella.af. OR nasopharyngealcarcinoma.af. OR nasopharyngeale.af. OR nasopharyngealelektroden.af. OR nasopharyngealem.af. OR nasopharyngealen.af. OR nasopharyngealer.af. OR nasopharyngeales.af. OR nasopharyngealis.af. OR nasopharyngeally.af. OR nasopharyngealpassages.af. OR nasopharyngealpodning.af. OR nasopharyngealt.af. OR nasopharyngeat.af. OR nasopharyngectomies.af. OR nasopharyngectomy.af. OR nasopharyngee.af. OR nasopharyngel.af. OR nasopharyngeoscopy.af. OR nasopharyngerl.af. OR nasopharynges.af. OR nasopharyngeum.af. OR nasopharyngeus.af. OR nasopharyngheal.af. OR nasopharyngial.af. OR nasopharyngien.af. OR nasopharyngienne.af. OR nasopharyngiens.af. OR nasopharyngioma.af. OR nasopharyngis.af. OR nasopharyngital.af. OR nasopharyngitidis.af. OR nasopharyngitis.af. OR nasopharyngitis'.af. OR nasopharyngitises.af. OR nasopharyngo.af. OR nasopharyngoal.af. OR nasopharyngobronchial.af. OR nasopharyngoendoscopy.af. OR nasopharyngofiberoscope.af. OR nasopharyngofiberscopy.af. OR nasopharyngogram.af. OR nasopharyngograms.af. OR nasopharyngography.af. OR nasopharyngolarygnoscope.af. OR nasopharyngolaryngeal.af. OR nasopharyngolaryngee.af. OR nasopharyngolaryngoscope.af. OR nasopharyngolaryngoscopes.af. OR nasopharyngolaryngoscopic.af. OR nasopharyngolaryngoscopie.af. OR nasopharyngolaryngoscopies.af. OR nasopharyngolaryngoscopy.af. OR nasopharyngolaryngovideoscope.af. OR nasopharyngolarynx.af. OR nasopharyngometry.af. OR nasopharyngoscope.af. OR nasopharyngoscopes.af. OR nasopharyngoscopic.af. OR nasopharyngoscopically.af. OR nasopharyngoscopies.af. OR nasopharyngoscopy.af. OR nasopharyngoskop.af. OR nasopharyngosopic.af. OR nasopharyngotis.af. OR nasopharyngral.af. OR nasopharyngs.af. OR nasopharyngscopy.af. OR nasopharyngtis.af. OR nasopharyngus.af. OR nasopharyngx.af. OR nasopharynlaryngoscope.af. OR nasopharynogoscopy.af. OR nasopharyns.af. OR nasopharynx.af. OR nasopharynx'.af. OR nasopharynx's.af. OR nasopharynx2.af. OR nasopharynxandnasopharyngeal.af. OR nasopharynxaspirat.af. OR nasopharynxbefall.af. OR nasopharynxduring.af. OR nasopharynxelektrode.af. OR nasopharynxes.af. OR nasopharynxkarcinomer.af. OR nasopharynxkarzinom.af. OR nasopharynxkarzinome.af. OR nasopharynxkarzinomen.af. OR nasopharynxkarzinompatienten.af. OR nasopharynxkarzinoms.af. OR nasopharynxmalignom.af. OR nasopharynxmalignome.af. OR nasopharynxprozessen.af. OR nasopharynxtuberkulos.af. OR nasopharynxtuberkulose.af. OR nasopharynxtumor.af. OR nasopharynxtumoren.af. OR nasopharynxtumors.af. OR nasopharyny.af. OR nasopharynyeal.af. OR nasopharynz.af.)) AND 20000:2020.(yr). | ("Streptococcus pneumoniae".af. OR (pneumococ.af. OR pneumococaal.af. OR pneumococal.af. OR pneumococc.af. OR pneumococca.af. OR pneumococcaemia.af. OR pneumococcaemic.af. OR pneumococcal.af. OR pneumococcal'.af. OR pneumococcall.af. OR pneumococcalmeningitis.af. OR pneumococcalpneumonia.af. OR pneumococcalvaccine.af. OR pneumococccal.af. OR pneumococcccal.af. OR pneumococcemia.af. OR pneumococcemias.af. OR pneumococcemic.af. OR pneumococcemie.af. OR pneumococcemies.af. OR pneumococchi.af. OR pneumococci.af. OR pneumococci's.af. OR pneumococcia.af. OR pneumococcic.af. OR pneumococcica.af. OR pneumococcicas.af. OR pneumococciche.af. OR pneumococcicidal.af. OR pneumococcicide.af. OR pneumococcico.af. OR pneumococcidal.af. OR pneumococcie.af. OR pneumococcies.af. OR pneumococcique.af. OR pneumococciques.af. OR pneumococcis.af. OR pneumococclea.af. OR pneumococco.af. OR pneumococcoal.af. OR pneumococcol.af. OR pneumococcosis.af. OR pneumococcosuria.af. OR pneumococcous.af. OR pneumococctyper.af. OR pneumococcu.af. OR pneumococcuria.af. OR pneumococcus.af. OR pneumococcus'.af. OR pneumococcus's.af. OR pneumococcus19f.af. OR pneumococcusbetegseg.af. OR pneumococcusclinical.af. OR pneumococcusfertozes.af. OR pneumococcusok.af. OR pneumococcusperitonitis.af. OR pneumococcusprevencio.af. OR pneumococcusuria.af. OR pneumococcusvakcina.af. OR pneumococcusvakcinacio.af. OR pneumococcuswere.af. OR pneumococcusx.af. OR pneumococcy.af. OR pneumococeal.af. OR pneumocochlea.af. OR pneumococi.af. OR pneumococic.af. OR pneumococica.af. OR pneumococicas.af. OR pneumococice.af. OR pneumococicos.af. OR pneumococilor.af. OR pneumococique.af. OR pneumococo.af. OR pneumocococal.af. OR pneumocococcal.af. OR pneumococos.af. OR pneumococul.af. OR pneumococus.af.)) AND ((resistan.af. OR resistanc.af. OR resistanca.af. OR resistancce.af. OR resistance.af. OR resistance'.af. OR resistance''.af. OR resistance's.af. OR resistance,.af. OR resistance1.af. OR resistance11.af. OR resistance14.af. OR resistance2.af. OR resistance3.af. OR resistance4.af. OR resistance6.af. OR resistance8.af. OR resistance9.af. OR resistanceamong.af. OR resistanceand.af. OR resistanceare.af. OR resistanceassociated.af. OR resistancebreaking.af. OR resistancecaliber.af. OR resistancecan.af. OR resistanced.af. OR resistancedetermining.af. OR resistancedomaincontaining.af. OR resistancedurability.af. OR resistanceesophageal.af. OR resistanceetween.af. OR resistanceevolution.af. OR resistancega.af. OR resistancein.af. OR resistanceindex.af. OR resistanceir.af. OR resistancekurven.af. OR resistancel.af. OR resistancelocuso.af. OR resistancemap.af. OR resistancemcr.af. OR resistancemediated.af. OR resistancemeter.af. OR resistancenutrition.af. OR resistanceof.af. OR resistanceopen.af. OR resistanceplus.af. OR resistancerate.af. OR resistancerelated.af. OR resistanceright.af. OR resistances.af. OR resistances'.af. OR resistancesim.af. OR resistancesubmitted.af. OR resistancesusceptibility.af. OR resistancethis.af. OR resistanceto.af. OR resistancetype.af. OR resistancevars.af. OR resistancewas.af. OR resistancewere.af. OR resistancewith.af. OR resistancex.af. OR resistancexcompliance.af. OR resistancexpert.af. OR resistancia.af. OR resistancies.af. OR resistanct.af. OR resistancto.af. OR resistancy.af. OR resistand.af. OR resistane.af. OR resistanec.af. OR resistaneza.af. OR resistanfce.af. OR resistange.af. OR resistanhe.af. OR resistanr.af. OR resistans.af. OR resistanse.af. OR resistant.af. OR resistant'.af. OR resistant''.af. OR resistant's.af. OR resistant,.af. OR resistant1.af. OR resistant1's.af. OR resistant11.af. OR resistant12.af. OR resistant2.af. OR resistant2,3.af. OR resistant3.af. OR resistant30.af. OR resistant4.af. OR resistant41.af. OR resistant5.af. OR resistant6.af. OR resistanta.af. OR resistantacid.af. OR resistantacinetobacter.af. OR resistantance.af. OR resistantassociated.af. OR resistantb.af. OR resistantc.af. OR resistantcases.af. OR resistantce.af. OR resistantcell.af. OR resistantcells.af. OR resistante.af. OR resistantenterococci.af. OR resistantenterococcus.af. OR resistantenterococcusdetected.af. OR resistantes.af. OR resistantescherichia.af. OR resistantescherichiacolifrom.af. OR resistantgastric.af. OR resistantgroup.af. OR resistanth.af. OR resistanthypertension.af. OR resistantiae.af. OR resistantin.af. OR resistantl.af. OR resistantlike.af. OR resistantlpr.af. OR resistantly.af. OR resistantm.af. OR resistantmalaria.af. OR resistantmedicago.af. OR resistantmgrbmutants.af. OR resistantmicroorganisms.af. OR resistantmycobacterium.af. OR resistantmycoplasma.af. OR resistantn.af. OR resistantneisseria.af. OR resistantnicotiana.af. OR resistantp.af. OR resistantpathogens.af. OR resistantpathogensidentified.af. OR resistantphenotype.af. OR resistantplants.af. OR resistantplus.af. OR resistantprimary.af. OR resistantproteus.af. OR resistantpseudomonas.af. OR resistantpsuedomonas.af. OR resistantr.af. OR resistants.af. OR resistantstaphylococcus.af. OR resistantstrains.af. OR resistantstreptococcus.af. OR resistanttb.af. OR resistantto.af. OR resistanttodegradationby.af. OR resistanttuberculosis.af. OR resistantu.af. OR resistantwomen.af. OR resistanz.af. OR resistanza.af. OR resistanzce.af. OR resistanzspektrum.af.) OR (susceptib.af. OR susceptibbility.af. OR susceptibble.af. OR susceptibe.af. OR susceptibel.af. OR susceptibiblity.af. OR susceptibie.af. OR susceptibiity.af. OR susceptibil.af. OR susceptibile.af. OR susceptibili.af. OR susceptibilidad.af. OR susceptibilidade.af. OR susceptibilidades.af. OR susceptibilies.af. OR susceptibilify.af. OR susceptibililly.af. OR susceptibililties.af. OR susceptibililty.af. OR susceptibilily.af. OR susceptibilit.af. OR susceptibilita.af. OR susceptibilitat.af. OR susceptibilitate.af. OR susceptibilitatea.af. OR susceptibilitatii.af. OR susceptibilite.af. OR susceptibilites.af. OR susceptibilities.af. OR susceptibilities'.af. OR susceptibilitities.af. OR susceptibilitity.af. OR susceptibilitiy.af. OR susceptibilitv.af. OR susceptibility.af. OR susceptibility'.af. OR susceptibility's.af. OR susceptibility'testing.af. OR susceptibility,.af. OR susceptibility0020might.af. OR susceptibility1.af. OR susceptibility16.af. OR susceptibility2.af. OR susceptibility5.af. OR susceptibilitygene.af. OR susceptibilityof.af. OR susceptibilityrelated.af. OR susceptibilitystatus.af. OR susceptibilityto.af. OR susceptibilitywas.af. OR susceptibilityweighted.af. OR susceptibiliy.af. OR susceptibillities.af. OR susceptibillty.af. OR susceptibilties.af. OR susceptibiltity.af. OR susceptibiltiy.af. OR susceptibilty.af. OR susceptibily.af. OR susceptibitity.af. OR susceptibity.af. OR susceptibiulity.af. OR susceptible.af. OR susceptible'.af. OR susceptible'p.af. OR susceptible1.af. OR susceptible3.af. OR susceptibleinfected.af. OR susceptiblel.af. OR susceptibleleucaena.af. OR susceptiblelycopersicon.af. OR susceptiblemycobacterium.af. OR susceptiblen.af. OR susceptibleness.af. OR susceptibles.af. OR susceptibles'.af. OR susceptiblet.af. OR susceptibleto.af. OR susceptiblexresistant.af. OR susceptibliity.af. OR susceptiblility.af. OR susceptiblilty.af. OR susceptiblities.af. OR susceptiblity.af. OR susceptibly.af.) OR (sensitiv.af. OR sensitiva.af. OR sensitivae.af. OR sensitivas.af. OR sensitivatat.af. OR sensitivation.af. OR sensitivdty.af. OR sensitive.af. OR sensitive'.af. OR sensitive''.af. OR sensitive's.af. OR sensitive1.af. OR sensitive2.af. OR sensitive21.af. OR sensitive23.af. OR sensitive23d.af. OR sensitive3.af. OR sensitive3a.af. OR sensitive4.af. OR sensitive5.af. OR sensitive51.af. OR sensitive51c.af. OR sensitive52.af. OR sensitive81.af. OR sensitive9.af. OR sensitivebiochemical.af. OR sensitivebut.af. OR sensitivecardiac.af. OR sensitived.af. OR sensitivedatura.af. OR sensitiveefflux.af. OR sensitivefsgs.af. OR sensitivehsd.af. OR sensitivelethal.af. OR sensitively.af. OR sensitivem.af. OR sensitivemess.af. OR sensitivemutants.af. OR sensitivemutations.af. OR sensitiven.af. OR sensitivend.af. OR sensitiveness.af. OR sensitivenested.af. OR sensitivepatient.af. OR sensitivepro.af. OR sensitiveprostate.af. OR sensitiver.af. OR sensitives.af. OR sensitives'.af. OR sensitivesensorial.af. OR sensitiveshibire.af. OR sensitiveslow.af. OR sensitivetest.af. OR sensitiveto.af. OR sensitivetoolsforevaluating.af. OR sensitivetu.af. OR sensitivety.af. OR sensitivi.af. OR sensitivi'.af. OR sensitividade.af. OR sensitivie.af. OR sensitiviertem.af. OR sensitivies.af. OR sensitiviity.af. OR sensitivily.af. OR sensitiviness.af. OR sensitiving.af. OR sensitivisation.af. OR sensitivisering.af. OR sensitivit.af. OR sensitivita.af. OR sensitivitas.af. OR sensitivitat.af. OR sensitivitats.af. OR sensitivitatsanalyse.af. OR sensitivitatsanderungen.af. OR sensitivitatsindex.af. OR sensitivitatssteigerung.af. OR sensitivitatsstudie.af. OR sensitivitatsstudien.af. OR sensitivitatstest.af. OR sensitivitatstestung.af. OR sensitivitatsvergleich.af. OR sensitivitatsverlust.af. OR sensitivite.af. OR sensitivites.af. OR sensitivitet.af. OR sensitivitets.af. OR sensitivitetsforvirring.af. OR sensitivitetskurser.af. OR sensitivitetstranas.af. OR sensitivitetstraning.af. OR sensitivitetstraningen.af. OR sensitivitetstrening.af. OR sensitivitiable.af. OR sensitivitied.af. OR sensitivities.af. OR sensitivities'.af. OR sensitivitiesor.af. OR sensitivitiness.af. OR sensitivitites.af. OR sensitivitities.af. OR sensitivitity.af. OR sensitivitive.af. OR sensitivitives.af. OR sensitivitivity.af. OR sensitivitiy.af. OR sensitivitty.af. OR sensitivitv.af. OR sensitivity.af. OR sensitivity'.af. OR sensitivity''.af. OR sensitivity'comt.af. OR sensitivity's.af. OR sensitivity,.af. OR sensitivity0.af. OR sensitivity1.af. OR sensitivity100.af. OR sensitivity26.af. OR sensitivity28.af. OR sensitivity2x2xk.af. OR sensitivity5.af. OR sensitivity60.af. OR sensitivityanalyses.af. OR sensitivityand.af. OR sensitivityc.af. OR sensitivitycalizationand.af. OR sensitivitycardiac.af. OR sensitivitycasecontrol.af. OR sensitivitycontrast.af. OR sensitivityfor.af. OR sensitivityfunction.af. OR sensitivityhits.af. OR sensitivityinconclusive.af. OR sensitivityincreased.af. OR sensitivitykij.af. OR sensitivitylimit.af. OR sensitivitymagnetic.af. OR sensitivitymult.af. OR sensitivityno.af. OR sensitivityof.af. OR sensitivitypowered.af. OR sensitivityr.af. OR sensitivitys.af. OR sensitivitysers.af. OR sensitivityspecificity.af. OR sensitivityspecificitydiagnostic.af. OR sensitivityspecificitypositive.af. OR sensitivitytesting.af. OR sensitivityto.af. OR sensitivitytroponin.af. OR sensitivityvery.af. OR sensitivitywarfarin.af. OR sensitivityxspecificity.af. OR sensitivityxstress.af. OR sensitivive.af. OR sensitiviy.af. OR sensitiviyy.af. OR sensitivization.af. OR sensitivized.af. OR sensitivly.af. OR sensitivnach.af. OR sensitivnaia.af. OR sensitivni.af. OR sensitivniia.af. OR sensitivnogo.af. OR sensitivnom.af. OR sensitivnosti.af. OR sensitivnyi.af. OR sensitivnym.af. OR sensitivnymi.af. OR sensitivo.af. OR sensitivomoteurs.af. OR sensitivomotor.af. OR sensitivomotora.af. OR sensitivomotoras.af. OR sensitivomotrice.af. OR sensitivomotrices.af. OR sensitivomotriz.af. OR sensitivos.af. OR sensitivovegetatif.af. OR sensitivovegetatives.af. OR sensitivrty.af. OR sensitivste.af. OR sensitivt.af. OR sensitivtities.af. OR sensitivtity.af. OR sensitivty.af. OR sensitivum.af. OR sensitivus.af. OR sensitivy.af. OR sensitivyt.af.)) AND (carriage.af. OR colonization.af. OR (invasiv.af. OR invasiva.af. OR invasivally.af. OR invasivas.af. OR invasivasui.af. OR invasive.af. OR invasive'.af. OR invasive''.af. OR invasive1.af. OR invasive1,2.af. OR invasive3.af. OR invasive3,4.af. OR invasiveacacia.af. OR invasiveapproach.af. OR invasivebladder.af. OR invasivebrain.af. OR invasivecardiac.af. OR invasivecollection.af. OR invasived.af. OR invasivediagnosis.af. OR invasivedoc.af. OR invasiveescherichia.af. OR invasiveive.af. OR invasiveless.af. OR invasivelesslessbenefit.af. OR invasivelly.af. OR invasively.af. OR invasively'.af. OR invasivem.af. OR invasivemethods.af. OR invasivemodality.af. OR invasivemole.af. OR invasiven.af. OR invasivencss.af. OR invasivene.af. OR invasivenes.af. OR invasiveness.af. OR invasiveness'.af. OR invasivenessdagger.af. OR invasivenesss.af. OR invasivenoninvasive.af. OR invasivenss.af. OR invasiveo.af. OR invasivep.af. OR invasivepossible.af. OR invasiver.af. OR invasiverespiratory.af. OR invasives.af. OR invasives'.af. OR invasivesess.af. OR invasivesness.af. OR invasivespeciesinfo.af. OR invasivesurgery.af. OR invasivetechniques.af. OR invasivetka.af. OR invasivetreatment.af. OR invasivetype.af. OR invasiveusing.af. OR invasivewhile.af. OR invasivi.af. OR invasividad.af. OR invasividade.af. OR invasiviness.af. OR invasivion.af. OR invasivita.af. OR invasivitat.af. OR invasivitatsreduktion.af. OR invasivite.af. OR invasivitet.af. OR invasivities.af. OR invasivity.af. OR invasivkardiologen.af. OR invasivkardiologie.af. OR invasivly.af. OR invasivness.af. OR invasivni.af. OR invasivnykh.af. OR invasivo.af. OR invasivos.af. OR invasivt.af. OR invasivum.af.) OR ("Blood".fs. OR blood.af. OR exp blood/) OR CSF.af. OR (cerebrospin.af. OR cerebrospina.af. OR cerebrospinaie.af. OR cerebrospinaiis.af. OR cerebrospinal.af. OR cerebrospinala.af. OR cerebrospinale.af. OR cerebrospinalen.af. OR cerebrospinaler.af. OR cerebrospinales.af. OR cerebrospinalfluessigkeit.af. OR cerebrospinalfluid.af. OR cerebrospinalflusigkeit.af. OR cerebrospinalflussigkeit.af. OR cerebrospinali.af. OR cerebrospinalis.af. OR cerebrospinalis1.af. OR cerebrospinalisban.af. OR cerebrospinalliquor.af. OR cerebrospinalmeningitt.af. OR cerebrospinalna.af. OR cerebrospinalnaho.af. OR cerebrospinalne.af. OR cerebrospinalneho.af. OR cerebrospinalni.af. OR cerebrospinalniho.af. OR cerebrospinalnim.af. OR cerebrospinalnog.af. OR cerebrospinalnoi.af. OR cerebrospinalnoj.af. OR cerebrospinalnom.af. OR cerebrospinals.af. OR cerebrospinalvaeske.af. OR cerebrospinalvaeskelaekage.af. OR cerebrospinalvaesken.af. OR cerebrospinalvaeskens.af. OR cerebrospinalvatska.af. OR cerebrospinalvatskan.af. OR cerebrospinla.af. OR cerebrospinmal.af. OR cerebrospino.af. OR cerebrospinous.af. OR cerebrospinsl.af. OR cerebrospinslnoj.af.) OR (nasopharyn.af. OR nasopharyneal.af. OR nasopharynectomy.af. OR nasopharynegal.af. OR nasopharynegeal.af. OR nasopharyng.af. OR nasopharyngaeal.af. OR nasopharyngael.af. OR nasopharyngaitis.af. OR nasopharyngal.af. OR nasopharynge.af. OR nasopharyngea.af. OR nasopharyngead.af. OR nasopharyngeal.af. OR nasopharyngeal'.af. OR nasopharyngealbordetella.af. OR nasopharyngealcarcinoma.af. OR nasopharyngeale.af. OR nasopharyngealelektroden.af. OR nasopharyngealem.af. OR nasopharyngealen.af. OR nasopharyngealer.af. OR nasopharyngeales.af. OR nasopharyngealis.af. OR nasopharyngeally.af. OR nasopharyngealpassages.af. OR nasopharyngealpodning.af. OR nasopharyngealt.af. OR nasopharyngeat.af. OR nasopharyngectomies.af. OR nasopharyngectomy.af. OR nasopharyngee.af. OR nasopharyngel.af. OR nasopharyngeoscopy.af. OR nasopharyngerl.af. OR nasopharynges.af. OR nasopharyngeum.af. OR nasopharyngeus.af. OR nasopharyngheal.af. OR nasopharyngial.af. OR nasopharyngien.af. OR nasopharyngienne.af. OR nasopharyngiens.af. OR nasopharyngioma.af. OR nasopharyngis.af. OR nasopharyngital.af. OR nasopharyngitidis.af. OR nasopharyngitis.af. OR nasopharyngitis'.af. OR nasopharyngitises.af. OR nasopharyngo.af. OR nasopharyngoal.af. OR nasopharyngobronchial.af. OR nasopharyngoendoscopy.af. OR nasopharyngofiberoscope.af. OR nasopharyngofiberscopy.af. OR nasopharyngogram.af. OR nasopharyngograms.af. OR nasopharyngography.af. OR nasopharyngolarygnoscope.af. OR nasopharyngolaryngeal.af. OR nasopharyngolaryngee.af. OR nasopharyngolaryngoscope.af. OR nasopharyngolaryngoscopes.af. OR nasopharyngolaryngoscopic.af. OR nasopharyngolaryngoscopie.af. OR nasopharyngolaryngoscopies.af. OR nasopharyngolaryngoscopy.af. OR nasopharyngolaryngovideoscope.af. OR nasopharyngolarynx.af. OR nasopharyngometry.af. OR nasopharyngoscope.af. OR nasopharyngoscopes.af. OR nasopharyngoscopic.af. OR nasopharyngoscopically.af. OR nasopharyngoscopies.af. OR nasopharyngoscopy.af. OR nasopharyngoskop.af. OR nasopharyngosopic.af. OR nasopharyngotis.af. OR nasopharyngral.af. OR nasopharyngs.af. OR nasopharyngscopy.af. OR nasopharyngtis.af. OR nasopharyngus.af. OR nasopharyngx.af. OR nasopharynlaryngoscope.af. OR nasopharynogoscopy.af. OR nasopharyns.af. OR nasopharynx.af. OR nasopharynx'.af. OR nasopharynx's.af. OR nasopharynx2.af. OR nasopharynxandnasopharyngeal.af. OR nasopharynxaspirat.af. OR nasopharynxbefall.af. OR nasopharynxduring.af. OR nasopharynxelektrode.af. OR nasopharynxes.af. OR nasopharynxkarcinomer.af. OR nasopharynxkarzinom.af. OR nasopharynxkarzinome.af. OR nasopharynxkarzinomen.af. OR nasopharynxkarzinompatienten.af. OR nasopharynxkarzinoms.af. OR nasopharynxmalignom.af. OR nasopharynxmalignome.af. OR nasopharynxprozessen.af. OR nasopharynxtuberkulos.af. OR nasopharynxtuberkulose.af. OR nasopharynxtumor.af. OR nasopharynxtumoren.af. OR nasopharynxtumors.af. OR nasopharyny.af. OR nasopharynyeal.af. OR nasopharynz.af.)) AND 2000:2020.(yr). |
| Cereda (2016)^1^ | ((("nutrition assessment"[MeSH Terms] OR ("nutrition"[All Fields] AND "assessment"[All Fields]) OR "nutrition assessment"[All Fields] OR ("mini"[All Fields] AND "nutritional"[All Fields] AND "assessment"[All Fields]) OR "mini nutritional assessment"[All Fields]) AND "mna"[All Fields]) AND (english[Language])) AND (("0000"[Date - Publication] : "2014/12/31"[Date - Publication])) | (((exp "nutrition assessment"/ OR (nutrition.af. AND assessment.af.) OR "nutrition assessment".af. OR (mini.af. AND nutritional.af. AND assessment.af.) OR "mini nutritional assessment".af.) AND mna.af.) AND (english.lg.)) AND 0000:2014.(yr). | (((exp "nutrition assessment"/ OR (nutrition.af. AND assessment.af.) OR "nutrition assessment".af. OR (mini.af. AND nutritional.af. AND assessment.af.) OR "mini nutritional assessment".af.) AND mna.af.) AND (english.lg.)) AND 0000:2014.(yr). |
| Christensen (2023)^1^ | (("Dermatitis, Atopic"[Mesh] OR "atopic dermatitis"[all fields] OR "dermatitis atopic"[all fields] OR "Eczema"[Mesh] OR "eczema"[all fields] OR "childhood eczema"[all fields] OR "infantile eczema"[all fields] OR "neurodermatitis"[all fields] OR "Neurodermatitis"[Mesh] OR "Besnier's prurigo"[all fields] OR ("Besnier's"[all fields] AND "prurigo"[all fields])) AND ("food allergy"[MeSH Terms] OR "food allergy"[all fields] OR "food sensitization"[all fields] OR "food provocation"[all fields] OR "food sensitivity"[all fields] OR "diet allergy"[all fields] OR "peanut allergy"[all fields] OR "nut allergy"[all fields] OR "cod allergy"[all fields] OR "milk allergy"[all fields] OR "egg allergy"[all fields] OR "fish allergy"[all fields] OR "shellfish allergy"[all fields] OR "wheat allergy"[all fields] OR "soybeans allergy"[all fields]) ) AND (("0000"[Date - Publication] : "2021/10/23"[Date - Publication])) | ((exp "atopic dermatitis"/ OR "atopic dermatitis".af. OR "dermatitis atopic".af. OR exp Eczema/ OR eczema.af. OR "childhood eczema".af. OR "infantile eczema".af. OR neurodermatitis.af. OR exp Neurodermatitis/ OR "Besnier's prurigo".af. OR (Besnier'.af. AND prurigo.af.)) AND (exp "food allergy"/ OR "food allergy".af. OR "food sensitization".af. OR "food provocation".af. OR "food sensitivity".af. OR "diet allergy".af. OR "peanut allergy".af. OR "nut allergy".af. OR "cod allergy".af. OR "milk allergy".af. OR "egg allergy".af. OR "fish allergy".af. OR "shellfish allergy".af. OR "wheat allergy".af. OR "soybeans allergy".af.)) AND 0000:2021.(yr). | ((exp "Dermatitis, Atopic"/ OR "atopic dermatitis".af. OR "dermatitis atopic".af. OR exp Eczema/ OR eczema.af. OR "childhood eczema".af. OR "infantile eczema".af. OR neurodermatitis.af. OR exp Neurodermatitis/ OR "Besnier's prurigo".af. OR (Besnier'.af. AND prurigo.af.)) AND (exp "food allergy"/ OR "food allergy".af. OR "food sensitization".af. OR "food provocation".af. OR "food sensitivity".af. OR "diet allergy".af. OR "peanut allergy".af. OR "nut allergy".af. OR "cod allergy".af. OR "milk allergy".af. OR "egg allergy".af. OR "fish allergy".af. OR "shellfish allergy".af. OR "wheat allergy".af. OR "soybeans allergy".af.)) AND 0000:2021.(yr). |
| Foley (2022)^2^ | (("multimorbidity"[MeSH Terms] OR "multimorbid*"[Title/Abstract] OR "multi-morbid*"[Title/Abstract] OR "multidisease*"[Title/Abstract] OR "multi-disease*"[Title/Abstract] OR "comorbidity"[MeSH Terms] OR "comorbid*"[Title/Abstract] OR "co-morbid*"[Title/Abstract] OR "chronic medication*"[Title/Abstract] OR "polypharma*"[Title/Abstract]) AND ("medication adherence"[MeSH Terms] OR "adheren*"[Title/Abstract] OR "nonadheren*"[Title/Abstract] OR "non-adheren*"[Title/Abstract] OR "non adheren*"[Title/Abstract] OR "complian*"[Title/Abstract] OR "noncomplian*"[Title/Abstract] OR "non-complian*"[Title/Abstract] OR "non complian*"[Title/Abstract]) )   - Publication/search dates stated in the paper: from January 2009 to April 2019 | ((exp 'multiple chronic conditions'/ OR multimorbid*.ti,ab. OR "multi-morbid*".ti,ab. OR multidisease*.ti,ab. OR "multi-disease*".ti,ab. OR (multiple adj2 (ill* or disease* or condition* or syndrom* or disorder*)).ab,ti. OR exp comorbidity/ OR comorbid*.ti,ab. OR "co-morbid*".ti,ab. OR (chronic adj2 (disease* or ill* or care or condition* or disorder* or health* or medication* or syndrom* or symptom*)).ti,ab. OR polypharma*.ti,ab.) AND (exp "medication compliance"/ OR "medication adherence".ti,ab OR adheren*.ti,ab. OR nonadheren*.ti,ab. OR "non-adheren*".ti,ab OR "non adheren*".ti,ab. OR complian*.ti,ab. OR noncomplian*.ti,ab. OR "non-complian*".ti,ab. OR "non complian*".ti,ab.)) AND 2009:2019.(yr). | ((exp multimorbidity/ OR multimorbid*.tw. OR "multi-morbid*".tw. OR multidisease*.tw. OR "multi-disease*".tw. OR exp comorbidity/ OR comorbid*.tw. OR "co-morbid*".tw. OR "chronic medication*".tw. OR polypharma*.tw.) AND (exp "medication adherence"/ OR adheren*.tw. OR nonadheren*.tw. OR "non-adheren*".tw. OR "non adheren*".tw. OR complian*.tw. OR noncomplian*.tw. OR "non-complian*".tw. OR "non complian*".tw.)) AND 2009:2019.(yr). |
| Gallego (2012)^1^ | ((antipsychotics AND (polypharmacy OR comedications OR coprescription OR concomitant OR cotreatment OR combination OR adjunctive)))   - Publication/search dates stated in the paper: between 1970 and May 2009 | ((antipsychotics AND (polypharmacy OR comedications OR coprescription OR concomitant OR cotreatment OR combination OR adjunctive ))).af. AND 1970:2009.(yr). | ((antipsychotics AND (polypharmacy OR comedications OR coprescription OR concomitant OR cotreatment OR combination OR adjunctive ))).af. AND 1970:2009.(yr). |
| Gong (2022)^2^ | ("Parkinson Disease"[MeSH Terms] OR ("Paralysis Agitans"[Title/Abstract] OR "PD"[Title/Abstract] OR "parkinson*"[Title/Abstract])) AND ("deglutition disorders"[MeSH Terms] OR ("dysphagia"[Title/Abstract] OR "deglutition disorder*"[Title/Abstract] OR "swallowing dysfunction"[Title/Abstract] OR "swallowing disorder"[Title/Abstract] OR "impaired swallowing"[Title/Abstract] OR "acataposis"[Title/Abstract] OR "swallow problem*"[Title/Abstract])) AND "0000" [Date - Publication]: "2022/06/28" [Date - Publication] | ("Paralysis Agitans" OR PD OR parkinson*).ti,ab,kw. AND (dysphagia OR "deglutition disorder*" OR "swallowing dysfunction" OR "swallowing disorder" OR "impaired swallowing" OR acataposis OR "swallow problem*").ti,ab,kw. AND 0000:2022.(yr). | (exp "Parkinson Disease"/ OR ("Paralysis Agitans" OR PD OR parkinson*).ti,ab,kw.) AND (exp "deglutition disorders"/ OR (dysphagia OR "deglutition disorder*" OR "swallowing dysfunction" OR "swallowing disorder" OR "impaired swallowing" OR acataposis OR "swallow problem*").ti,ab,kw.) AND 0000:2022.(yr). |
| Hu (2017)^1^ | ("Helicobacter pylori"[All Fields] OR "h pylori"[All Fields] OR "Hp"[All Fields]) AND ("recurrence"[All Fields] OR "recrudescence"[All Fields] OR "reinfection"[All Fields] OR "recurrent"[All Fields] OR "recurred"[All Fields] OR "re-infect*"[All Fields] OR "relapse*"[All Fields]) AND "English"[Language] NOT (("Animals"[MeSH Terms] OR "Plants"[MeSH Terms]) NOT "Humans"[MeSH Terms]) AND "1983/06/01" [Date - Publication]: "2017/03/01" [Date - Publication] | ("Helicobacter pylori".af. OR "h pylori".af. OR Hp.af.) AND (recurrence.af. OR recrudescence.af. OR reinfection.af. OR recurrent.af. OR recurred.af. OR "re-infect*".af. OR relapse*.af.) AND english.lg NOT ((exp "animal"/ or exp "plant") not "human"/) AND 1983:2017.(yr). | ("Helicobacter pylori".af. OR "h pylori".af. OR Hp.af.) AND (recurrence.af. OR recrudescence.af. OR reinfection.af. OR recurrent.af. OR recurred.af. OR "re-infect*".af. OR relapse*.af.) AND english.lg NOT ((exp "Animals"/ or exp "Plants") not "Humans"/) AND 1983:2017.(yr). |
| Ibrahim (2017)^1^ | ((helicobacter pylori OR campylobacter pylori) AND (incidence OR prevalence OR "risk factors" OR determinants OR (lifestyle OR lifestyles) OR (tobacco OR smoking OR cigarette OR smoke) OR ("dietary pattern" OR "dietary patterns" OR "eating pattern" OR "eating patterns" OR "food pattern" OR "food patterns") OR (diet OR fruits OR vegetables OR antioxidants) OR (alcohol OR drinking) OR (salt OR salted OR nacl OR "sodium chloride" OR sodium OR "processed meat" OR "salt preserved foods" OR "smoked food") OR coffee OR tea OR (obes* OR "body mass index" OR bmi OR overweight) OR (diabetes OR glycemia OR hyperglycemia OR "impaired fasting glucose" OR IFG OR "impaired glucose tolerance") OR (crowding OR overcrowding) OR ("socioeconomic status" OR "socioeconomic level" OR ses OR "blood type" OR "blood group" OR "lewis antigen")) NOT (animals[mh] NOT humans[mh]))   - Publication/search dates stated in the paper: from inception to September 2015 | (("helicobacter pylori" OR "campylobacter pylori" ) AND (incidence OR prevalence OR "risk factors" OR determinants OR (lifestyle OR lifestyles ) OR (tobacco OR smoking OR cigarette OR smoke ) OR ("dietary pattern" OR "dietary patterns" OR "eating pattern" OR "eating patterns" OR "food pattern" OR "food patterns" ) OR (diet OR fruits OR vegetables OR antioxidants ) OR (alcohol OR drinking ) OR (salt OR salted OR nacl OR "sodium chloride" OR sodium OR "processed meat" OR "salt preserved foods" OR "smoked food" ) OR coffee OR tea OR (obes* OR "body mass index" OR bmi OR overweight ) OR (diabetes OR glycemia OR hyperglycemia OR "impaired fasting glucose" OR IFG OR "impaired glucose tolerance" ) OR (crowding OR overcrowding ) OR ("socioeconomic status" OR "socioeconomic level" OR ses OR "blood type" OR "blood group" OR "lewis antigen" )) NOT (exp animal/ NOT exp human/)) AND 0000:2015.(yr). | (("helicobacter pylori" OR "campylobacter pylori" ) AND (incidence OR prevalence OR "risk factors" OR determinants OR (lifestyle OR lifestyles ) OR (tobacco OR smoking OR cigarette OR smoke ) OR ("dietary pattern" OR "dietary patterns" OR "eating pattern" OR "eating patterns" OR "food pattern" OR "food patterns" ) OR (diet OR fruits OR vegetables OR antioxidants ) OR (alcohol OR drinking ) OR (salt OR salted OR nacl OR "sodium chloride" OR sodium OR "processed meat" OR "salt preserved foods" OR "smoked food" ) OR coffee OR tea OR (obes* OR "body mass index" OR bmi OR overweight ) OR (diabetes OR glycemia OR hyperglycemia OR "impaired fasting glucose" OR IFG OR "impaired glucose tolerance" ) OR (crowding OR overcrowding ) OR ("socioeconomic status" OR "socioeconomic level" OR ses OR "blood type" OR "blood group" OR "lewis antigen" )) NOT (exp animals/ NOT exp humans/)) AND 0000:2015.(yr). |
| Kosmopoulos (2023)^3^ | (Constrictive OR Constriction) AND Pericarditis   - Publication/search dates stated in the paper: from 1939 to 2021 | (Constrictive or Pericarditis or constrictive pericarditis).ti,ab. AND 1939:2021.(yr). | (Constrictive OR Constriction) AND Pericarditis  AND 1939:2021.(yr). |
| Lopes (2021)^1^ | ((hypomineralization OR hypomineralisation OR hypomineralized OR hypomineralized OR hypoplasia OR demarcated OR opacities OR MIH OR cheese molars) AND (survey OR questionnaire OR cross-sectional OR prevalence OR frequency OR population OR sample OR sampling) AND (molar OR molars OR incisors))   - Publication/search dates stated in the paper: up to July 2021 | ((hypomineralization OR hypomineralisation OR hypomineralized OR hypomineralized OR hypoplasia OR demarcated OR opacities OR MIH OR "cheese molars" ) AND (survey OR questionnaire OR cross-sectional OR prevalence OR frequency OR population OR sample OR sampling ) AND (molar OR molars OR incisors )) AND 0000:2021.(yr). | ((hypomineralization OR hypomineralisation OR hypomineralized OR hypomineralized OR hypoplasia OR demarcated OR opacities OR MIH OR "cheese molars" ) AND (survey OR questionnaire OR cross-sectional OR prevalence OR frequency OR population OR sample OR sampling ) AND (molar OR molars OR incisors )) AND 0000:2021.(yr). |
| Lu (2023)^4^ | ("heart"[Title/Abstract] OR "cardiac"[Title/Abstract] OR "cardiovascular"[Title/Abstract] OR "cardiotoxicity"[Title/Abstract] OR ("myocardial infarction"[Title/Abstract] OR "ischemia"[Title/Abstract] OR "coronary artery"[Title/Abstract] OR "acute coronary syndrome"[Title/Abstract] OR "coronary vasospasm"[Title/Abstract] OR "chest pain"[Title/Abstract] OR "angina"[Title/Abstract] OR "syncope"[Title/Abstract] OR "dyspnea"[Title/Abstract] OR "myocardium"[Title/Abstract] OR "palpitation"[Title/Abstract] OR "ventricular dysfunction"[Title/Abstract] OR "systolic dysfunction"[Title/Abstract] OR "heart failure"[Title/Abstract] OR "LVEF"[Title/Abstract] OR "atrioventricular block"[Title/Abstract] OR "conduction disorder"[Title/Abstract] OR "bundle branch block"[Title/Abstract] OR "arrhythmia"[Title/Abstract] OR "tachycardia"[Title/Abstract] OR "bradycardia"[Title/Abstract] OR "atrial fibrillation"[Title/Abstract] OR "ventricular fibrillation"[Title/Abstract] OR "repolarization"[Title/Abstract] OR "atrial flutter"[Title/Abstract] OR "ECG"[Title/Abstract] OR "pericardial effusion"[Title/Abstract] OR "pericarditis"[Title/Abstract] OR "pericardial tamponade"[Title/Abstract] OR "cardiac myositis"[Title/Abstract] OR "myocarditis"[Title/Abstract] OR "cardiomyopathy"[Title/Abstract] OR "heart-sac"[Title/Abstract] OR "cardiac arrest"[Title/Abstract] OR "sudden death"[Title/Abstract] OR "cardiogenic shock"[Title/Abstract] OR "regurgitation"[Title/Abstract] OR "echocardiography"[Title/Abstract] OR "cardiac biomarkers"[Title/Abstract] OR "troponin"[Title/Abstract] OR "enzyme"[Title/Abstract] OR "death"[Title/Abstract])) AND ("fluoropyridine"[Title/Abstract] OR "fluoropyrimidine"[Title/Abstract] OR "5 fluorouracil"[Title/Abstract] OR "5-FU"[Title/Abstract] OR "fluorouracil"[Title/Abstract] OR "capecitabine"[Title/Abstract] OR "xeloda"[Title/Abstract] OR "tegafur"[Title/Abstract] OR "S-1"[Title/Abstract] OR "teysuno"[Title/Abstract] OR "UFT"[Title/Abstract] OR "carmofur"[Title/Abstract] OR "TAS-102"[Title/Abstract] OR "lonsurf"[Title/Abstract] OR "ftd tpi"[Title/Abstract] OR "trifluridine"[Title/Abstract] OR "tipiracil"[Title/Abstract])   - Publication/search dates stated in the paper: from the establishment of each database/website to 31 October 2022 | (heart.ab OR cardiac.ab OR cardiovascular.ab OR cardiotoxicity.ab OR ((((((((((((((((((((((myocardial.ab AND infarction.ab OR ischemia.ab OR coronary.ab) AND artery.ab OR acute.ab) AND coronary.ab AND syndrome.ab OR coronary.ab) AND vasospasm.ab OR chest.ab) AND pain.ab OR angina.ab OR syncope.ab OR dyspnea.ab OR myocardiumor.ab) AND palpitation.ab OR ventricular.ab) AND dysfunction.ab OR systolic.ab) AND dysfunction.ab OR heart.ab) AND failure.ab OR lvef.ab OR atrioventricular.ab) AND block.ab OR conduction.ab) AND disorder.ab OR bundle.ab) AND branch.ab AND block.ab OR arrhythmia.ab OR tachycardia.ab OR bradycardia.ab OR atrial.ab) AND fibrillation.ab OR ventricular.ab) AND fibrillation.ab OR repolarization.ab OR atrial.ab) AND flutter.ab OR ecg.ab OR pericardial.ab) AND effusion.ab OR pericarditis.ab OR pericardial.ab) AND tamponade.ab OR cardiac.ab) AND myositis.ab OR myocarditis.ab OR cardiomyopathy.ab OR 'heart sac'.ab OR cardiac.ab) AND arrest.ab OR sudden.ab) AND death.ab OR cardiogenic.ab) AND shock.ab OR regurgitation.ab OR echocardiography.ab OR cardiac.ab) AND biomarkers.ab) OR troponin.ab OR enzyme.ab OR death.ab) AND (fluoropyridine.ab OR fluoropyrimidine.ab OR '5 fluorouracil'.ab OR '5 fu'.ab OR fluorouracil.ab OR capecitabine.ab OR xeloda.ab OR tegafur.ab OR 's 1'.ab OR teysuno.ab OR uft.ab OR carmofur.ab OR 'tas 102'.ab OR lonsurf.ab OR ftd.ab OR tpi.ab OR trifluridine.ab OR tipiracil.ab) AND 0000:2022.(yr). | (heart OR cardiac OR cardiovascular OR cardiotoxicity OR ("myocardial infarction" OR ischemia OR "coronary artery" OR "acute coronary syndrome" OR "coronary vasospasm" OR "chest pain" OR angina OR syncope OR dyspnea OR myocardium OR palpitation OR "ventricular dysfunction" OR "systolic dysfunction" OR "heart failure" OR LVEF OR "atrioventricular block" OR "conduction disorder" OR "bundle branch block" OR arrhythmia OR tachycardia OR bradycardia OR "atrial fibrillation" OR "ventricular fibrillation" OR repolarization OR "atrial flutter" OR ECG OR "pericardial effusion" OR pericarditis OR "pericardial tamponade" OR "cardiac myositis" OR myocarditis OR cardiomyopathy OR heart-sac OR "cardiac arrest" OR "sudden death" OR "cardiogenic shock" OR regurgitation OR echocardiography OR "cardiac biomarkers" OR troponin OR enzyme OR death)).ab AND (fluoropyridine OR fluoropyrimidine OR "5 fluorouracil" OR 5-FU OR fluorouracil OR capecitabine OR xeloda OR tegafur OR S-1 OR teysuno OR UFT OR carmofur OR TAS-102 OR lonsurf OR "ftd tpi" OR trifluridine OR tipiracil).ab AND 0000:2022.(yr). |
| Meng (2022)^2^ | ((((Internet[Title] OR digital[Title] OR screen[Title] OR cyber*[Title] OR net[Title] OR online[Title] OR media[Title] OR electronic device*[Title] OR electronic gadgets[Title] OR computer[Title] OR mobile[Title] OR phone[Title] OR smartphone[Title] OR television[Title] OR TV[Title] OR video[Title] OR Facebook[Title] OR game[Title] OR gaming[Title])) AND (addict*[Title] OR use[Title] OR dependen*[Title] OR overuse[Title] OR abuse[Title] OR disorder[Title] OR excessive[Title] OR effects[Title] OR habits[Title] OR misuse[Title] OR pathological[Title] OR problem*[Title] OR compulsive[Title] OR heavy[Title])) AND (prevalence[Title/Abstract] OR survey[Title/Abstract] OR rate[Title/Abstract] OR scale[Title/Abstract] OR screening[Title/Abstract] OR situation[Title/Abstract] OR epidemic[Title/Abstract] OR epidemiological[Title/Abstract] OR occurrence[Title/Abstract] OR investigation[Title/Abstract]) )   - Publication/search dates stated in the paper: published before October 31, 2021 | ((Internet or digital or screen or cyber* or net or online or media or "electronic device*" or "electronic gadgets" or computer or mobile or phone or smartphone or television or TV or video or Facebook or game or gaming) and (addict* or "use" or dependen* or overuse or abuse or disorder or excessive or effects or habits or misuse or pathological or problem* or compulsive or heavy)).ti. and (prevalence or survey or rate or scale or screening or situation or epidemic or epidemiological or occurrence or investigation).ti,ab and 0000:2021.(yr). | ((Internet or digital or screen or cyber* or net or online or media or "electronic device*" or "electronic gadgets" or computer or mobile or phone or smartphone or television or TV or video or Facebook or game or gaming) and (addict* or "use" or dependen* or overuse or abuse or disorder or excessive or effects or habits or misuse or pathological or problem* or compulsive or heavy)).ti. and (prevalence or survey or rate or scale or screening or situn or epidemic or epidemiological or occurrence or inven).ti,ab,kw. and 0000:2021.(yr). |
| Raoofi (2023)^1^ | ("infection cross"[Title] OR "cross infections"[Title] OR "healthcare associated infections"[Title] OR "healthcare associated infection"[Title] OR "health care associated infection"[Title] OR "health care associated infections"[Title] OR "hospital infection"[Title] OR "infections hospital"[Title] OR "nosocomial infection"[Title] OR "nosocomial infections"[Title] OR "hospital infections"[Title])   - Publication/search dates stated in the paper: between 2000 and June 2021 | ("infection cross".ti. OR "cross infections".ti. OR "healthcare associated infections".ti. OR "healthcare associated infection".ti. OR "health care associated infection".ti. OR "health care associated infections".ti. OR "hospital infection".ti. OR "infections hospital".ti. OR "nosocomial infection".ti. OR "nosocomial infections".ti. OR "hospital infections".ti.) AND 2000:2021.(yr). | ("infection cross".ti. OR "cross infections".ti. OR "healthcare associated infections".ti. OR "healthcare associated infection".ti. OR "health care associated infection".ti. OR "health care associated infections".ti. OR "hospital infection".ti. OR "infections hospital".ti. OR "nosocomial infection".ti. OR "nosocomial infections".ti. OR "hospital infections".ti.) AND 2000:2021.(yr). |
| Singh (2022)^2^ | (“Hepatitis B” OR HBV OR “Hepatitis C” OR HCV OR “Hepatitis B” [MeSH Terms] OR “Hepatitis B Antigens” [MeSH Terms] OR “Hepatitis B Antibodies” [MeSH Terms] OR “Hepatitis B virus” [MeSH Terms] OR “Hepatitis C” [MeSH Terms] OR “Hepatitis C Antibodies” [MeSH Terms] OR “Hepatitis C Antigens” [MeSH Terms])  AND  (Patient [Text Word] OR Patients [Text Word] OR Outpatient [Text Word] OR Outpatients [Text Word] OR Patients [MeSH Terms] OR “Homebound person” [Text Word] OR “Homebound persons” [Text Word] OR “Homebound persons” [MeSH Terms] OR “Disabled person” [Text Word] OR “Disabled persons” [Text Word] OR “Disabled persons” [MeSH Terms] OR ”Health facility” OR “Health facilities” OR “Health facilities” [MeSH Terms] OR Hospital [Text Word] OR Hospitals [Text Word] OR Hospitals [MeSH Terms] OR “Home care agency” [Text Word] OR “Home care agencies” [Text Word] OR “Home care agencies” [MeSH Terms] OR “Voluntary health agencies” [MeSH Terms] OR “Health personnel” [MeSH Terms] OR “Health service” [Text Word] OR “Health services” [Text Word] OR “Health services” [MeSH Terms] OR “Health care associated” OR “Healthcare associated” OR “Iatrogenic” OR “Hospital acquired” OR “Cross infection” OR Nosocomial OR “Iatrogenic disease” [MeSH Terms] OR “Healthcare associated infections” [Text Word] OR “Associated infection, healthcare” [Text Word] OR “Associated infections, healthcare” OR “Healthcare associated infection”[Text Word] OR “Infection, Healthcare Associated” [Text Word] OR “Infections, Healthcare Associated” [Text Word] OR “Infection, Cross” [Text Word] OR “Health care associated infections” [Text Word] OR “Health care associated infection” [Text Word] OR “Hospital infections” [Text Word] OR “Hospital infection” [Text Word] OR “Infection, hospital” [Text Word] OR “Infections, hospital” [Text Word] OR “Infection, nosocomial” [Text Word] OR “Infections, nosocomial” [Text Word] OR “Nosocomial infection” [Text Word] OR “Nosocomial Infections” [Text Word])  AND  (Outbreak [Text Word] OR Outbreaks [Text Word] OR “Disease Outbreaks” [MeSH Terms] OR “Cluster analysis” [MeSH Terms] OR “Cluster” OR “Clusters” OR Transmission [Text Word] OR “Disease transmission, Infectious” [MeSH Terms])   - Publication/search dates stated in the paper: January 2006 to September   2021 | ("Hepatitis B" OR HBV OR "Hepatitis C" OR HCV OR exp "Hepatitis B"/ OR exp "Hepatitis B Antigen"/ OR exp "Hepatitis B Antibody"/ OR exp "Hepatitis B virus"/ OR exp "Hepatitis C"/ OR exp "Hepatitis C Antibodiy"/ OR exp "Hepatitis C Antigen"/ OR exp "Hepatitis C Virus"/) AND (Patient.ti,ab OR Patients.ti,ab OR Outpatient.ti,ab OR Outpatients.ti,ab OR "Homebound patient".ti,ab OR "Homebound patient".ti,ab OR "Disabled person".ti,ab OR "Disabled persons".ti,ab OR "Health care facility".ti,ab OR "Health care facilities".ti,ab OR Hospital.ti,ab OR Hospitals.ti,ab OR "Home care agency".ti,ab OR "Home care agencies".ti,ab OR "Home care".ti,ab OR "health service".ti,ab OR "health services".ti,ab OR "Health care associated infections".ti,ab OR "Cross infection".ti,ab OR "Hospital infection".ti,ab OR "Nosocomial infection".ti,ab OR "Health care facility" OR "Health care facilities" OR "Healthcare associated infection" OR "Hospital acquired" OR "Cross infection" OR "Nosocomial" OR exp "Nosocomial transmission"/ OR exp "patient"/ OR "disabled person"/ OR "mental disease"/ OR "mental deficiency"/ OR *"Health care facility"/ OR *"hospital"/ OR exp "Hospice" OR *"Health service"/ OR *"Health care personnel"/ OR "Iatrogenic disease"/) AND (outbreak.ti,ab OR outbreaks.ti,ab OR transmission.ti,ab OR cluster OR clusters OR "disease outbreak" OR exp "Cluster analysis"/ OR exp "Disease transmission"/) AND 2006:2021.(yr). | ("Hepatitis B" OR HBV OR "Hepatitis C" OR HCV OR exp "Hepatitis B"/ OR exp "Hepatitis B Antigens"/ OR exp "Hepatitis B Antibodies"/ OR exp "Hepatitis B virus"/ OR exp "Hepatitis C"/ OR exp "Hepatitis C Antibodies"/ OR exp "Hepatitis C Antigens"/)  AND (Patient.mp. OR Patients.mp. OR Outpatient.mp. OR Outpatients.mp. OR exp Patients/ OR "Homebound person".mp. OR "Homebound persons".mp. OR exp "Homebound persons"/ OR "Disabled person".mp. OR "Disabled persons".mp. OR exp "Disabled persons"/ OR "Health facility" OR "Health facilities" OR exp "Health facilities"/ OR Hospital.mp. OR Hospitals.mp. OR exp Hospitals/ OR "Home care agency".mp. OR "Home care agencies".mp. OR exp "Home care agencies"/ OR exp "Voluntary health agencies"/ OR exp "Health personnel"/ OR "Health service".mp. OR "Health services".mp. OR exp "Health services"/ OR "Health care associated" OR "Healthcare associated" OR Iatrogenic OR "Hospital acquired" OR "Cross infection" OR Nosocomial OR exp "Iatrogenic disease"/ OR "Healthcare associated infections".mp. OR "Associated infection, healthcare".mp. OR "Associated infections, healthcare" OR "Healthcare associated infection".mp. OR "Infection, Healthcare Associated".mp. OR "Infections, Healthcare Associated".mp. OR "Infection, Cross".mp. OR "Health care associated infections".mp. OR "Health care associated infection".mp. OR "Hospital infections".mp. OR "Hospital infection".mp. OR "Infection, hospital".mp. OR "Infections, hospital".mp. OR "Infection, nosocomial".mp. OR "Infections, nosocomial".mp. OR "Nosocomial infection".mp. OR "Nosocomial Infections".mp.)  AND (Outbreak.mp. OR Outbreaks.mp. OR exp "Disease Outbreaks"/ OR exp "Cluster analysis"/ OR Cluster OR Clusters OR Transmission.mp. OR exp "Disease transmission, Infectious"/) AND 2006:2021.(yr). |
| Ten Cate (2023)^1^ | (((((("isolated pulmonary embolism") OR "isolated PE") OR "PE without DVT" OR "without concomitant deep vein thrombosis") OR "concomitant DVT") OR "without peripheral VTE") OR "DVT-associated PE") OR "concurrent deep vein thrombosis")   - Publication/search dates stated in the paper: until January 27 2023 | ("isolated pulmonary embolism" OR "isolated PE" OR "PE without DVT" OR "without concomitant deep vein thrombosis" OR "concomitant DVT" OR "without peripheral VTE" OR "DVT-associated PE" OR "concurrent deep vein thrombosis") AND 0000:2023.(yr). | ("isolated pulmonary embolism" OR "isolated PE" OR "PE without DVT" OR "without concomitant deep vein thrombosis" OR "concomitant DVT" OR "without peripheral VTE" OR "DVT-associated PE" OR "concurrent deep vein thrombosis") AND 0000:2023.(yr). |
| Ting (2023)^1^ | ((("Trauma" OR "Injury*" OR "Post injury" OR "Post-injury") AND ("Multiple organ failure" OR "Organ failure" OR "MOF" OR "Organ dysfunction" OR "Multiple organ dysfunction syndrome" OR "MOD" or "MODS") AND ("Epidemiology*" OR "Incidence*" OR "Predict*" OR "Prediction model" OR "Outcome" OR "Sequelae" OR "Risk factor" OR "Mortality" OR "Prevalence")) AND (eng[Language] OR ger[Language]))   - Publication/search dates stated in the paper: from 1977 onwards; a search was performed on 9 December 2022 | ((Trauma or Injury* or "Post injury" or Post-injury) and ("Multiple organ failure" or "Organ failure" or MOF or "Organ dysfunction" or "Multiple organ dysfunction syndrome" or MOD or MODS) and (Epidemiology* or Incidence* or Predict* or "Prediction model" or Outcome or Sequelae or "Risk factor" or Mortality or Prevalence)) and (english or german).lg. and 1977:2022.(yr). | ((Trauma or Injury* or "Post injury" or Post-injury) and ("Multiple organ failure" or "Organ failure" or MOF or "Organ dysfunction" or "Multiple organ dysfunction syndrome" or MOD or MODS) and (Epidemiology* or Incidence* or Predict* or "Prediction model" or Outcome or Sequelae or "Risk factor" or Mortality or Prevalence)) and (english or german).lg. and 1977:2022.(yr). |

1 = search strategy was translated from MEDLINE(PubMed) for both Embase and MEDLINE(R) ALL Ovid
2 = search strategy was translated from Embase.com to Embase Ovid, while search strategy for MEDLINE(R) ALL was translated from MEDLINE(PubMed)
3 = search strategy for Embase Ovid was reproduced, while search strategy for MEDLINE(R) ALL was translated from MEDLINE(PubMed)
4 = search strategy was translated from Embase.com to Embase Ovid, while search strategy for MEDLINE(R) ALL was reproduced
